# SepG coordinates sporulation-specific cell division and nucleoid organization in *Streptomyces coelicolor*

**DOI:** 10.1098/rsob.150164

**Published:** 2016-04-06

**Authors:** Le Zhang, Joost Willemse, Dennis Claessen, Gilles P. van Wezel

**Affiliations:** Molecular Biotechnology, Institute of Biology, Leiden University, PO Box 9505, 2300RA Leiden, The Netherlands

**Keywords:** cell division control, development, FtsZ, SsgA-like proteins

## Abstract

Bacterial cell division is a highly complex process that requires tight coordination between septum formation and chromosome replication and segregation. In bacteria that divide by binary fission a single septum is formed at mid-cell, a process that is coordinated by the conserved cell division scaffold protein FtsZ. In contrast, during sporulation-specific cell division in streptomycetes, up to a hundred rings of FtsZ (Z rings) are produced almost simultaneously, dividing the multinucleoid aerial hyphae into long chains of unigenomic spores. This involves the active recruitment of FtsZ by the SsgB protein, and at the same time requires sophisticated systems to regulate chromosome dynamics. Here, we show that SepG is required for the onset of sporulation and acts by ensuring that SsgB is localized to future septum sites. Förster resonance energy transfer imaging suggests direct interaction between SepG and SsgB. The beta-lactamase reporter system showed that SepG is a transmembrane protein with its central domain oriented towards the cytoplasm. Without SepG, SsgB fails to localize properly, consistent with a crucial role for SepG in the membrane localization of the SsgB–FtsZ complex. While SsgB remains associated with FtsZ, SepG re-localizes to the (pre)spore periphery. Expanded doughnut-shaped nucleoids are formed in *sepG* null mutants, suggesting that SepG is required for nucleoid compaction. Taken together, our work shows that SepG, encoded by one of the last genes in the conserved *dcw* cluster of cell division and cell-wall-related genes in Gram-positive bacteria whose function was still largely unresolved*,* coordinates septum synthesis and chromosome organization in *Streptomyces*.

## 1. Introduction

Most unicellular bacteria grow and divide by binary fission, which involves an increase in cell length, chromosome replication and segregation, and septum formation, eventually resulting in two daughter cells that each inherits a single copy of the chromosome. Chromosome organization and segregation are tightly coordinated with the spatial and temporal initiation of cell division. The prokaryotic cell division scaffold is formed by the tubulin homologue FtsZ [1], which forms a contractile ring (or Z ring) that mediates recruitment of the cell division machinery to the mid-cell position (reviewed in [2,3]). In unicellular bacteria, septum-site localization and Z-ring stabilization is mediated by a number of proteins, including FtsA and ZipA [4–6], ZapA [7] and SepF [8,9]. The process of Z-ring (dis-)assembly during division is actively controlled (reviewed in [10]).

Streptomycetes are filamentous Gram-positive soil bacteria that have a complex multicellular life cycle [11,12], and produce over 60% of all known antibiotics and many other bioactive natural products [13,14]. Expression of these natural products is typically coordinated with the onset of spore development [15]. The vegetative mycelium consists of syncytial cells separated by widely spaced crosswalls [16]. During sporulation-specific cell division, FtsZ initially assembles in long filaments in the aerial hyphae, then as regular foci, to finally form a ladder of Z rings [17]. Eventually, cytokinesis results in long chains of spores, following a complex process of coordinated cell division and DNA segregation [18,19]. While *ftsZ* null mutants fail to produce septa and hence do not sporulate, cell division is not essential for growth of *Streptomyces*, which provides a unique system for the study of this process [19,20].

The mycelial lifestyle of streptomycetes imposes specific requirements for cell division control, in particular owing to the lack of a defined mid-cell position and the synchronous formation of multiple septa. The canonical model for the control of Z-ring formation involves the action of negative control systems such as Min, which prevents Z-ring assembly at the cell poles [21,22], and nucleoid occlusion, which prevents formation of the Z ring over non-segregated chromosomes [23–26]. However, in *Streptomyces,* septum-site localization is positively controlled, via the recruitment of FtsZ by SsgB, which in turn depends on the action of SsgA [27]. At the onset of sporulation-specific cell division, SsgA and SsgB briefly interact, but the precise role of SsgA in the recruitment of SsgB is still not well understood. The SsgA-like proteins are proteins that uniquely occur in actinomycetes [28,29]. This different mode of division control probably explains the absence of direct homologues of Min and Noc proteins and of the canonical Z-ring anchoring proteins. SepF and DivIVA are rare examples of cell division control proteins shared between *Streptomyces* and other bacteria, and of these, DivIVA is not involved in the control of division, but is instead required for driving apical (tip) extension and growth [30]. The concept of positive control of cell division is more widespread in bacteria, as it was also found in *Myxococcus xanthus,* where the ParA-like protein PomZ recruits FtsZ [31], whereas the recent discovery that *Bacillus* cells divide at mid-cell in the absence of Min and Noc may be explained by a positive control-like mechanism, although there the mechanism of septum positioning is unknown [32].

With the recruitment of FtsZ by SsgB, another important question is left unaddressed, namely how the highly symmetrical spacing between the sporulation septa is achieved; in other words, how is the localization of SsgB itself orchestrated? We previously suggested that the regularly spaced nucleoids might provide the boundaries for Z-ring assembly, in analogy with a nucleoid-occlusion mechanism [27]. One possible candidate is the conserved *ylmG* gene, which lies in the conserved *dcw* cluster for genes involved in division and cell-wall synthesis, and in-between *sepF* and *divIVA*. In firmicutes, SepF [8,9] and DivIVA [33] play an important role in the control of cell division in many Gram-positive bacteria, suggesting that SepG may also relate to the control of division. YlmG is especially found in actinobacteria, firmicutes and cyanobacteria, as well as in chloroplasts of photosynthetic eukaryotes. YlmG has been assigned to the YggT family of proteins (Pfam PF02325), which are named after *Escherichia coli* YggT and are a highly diverse family of membrane proteins found in bacteria and plastids [34]. Functional overlap between YlmG and the osmoregulation-related YggT is unlikely because of the lack of relevant sequence similarity and phylogenetic context. It was previously shown that the YlmG orthologue AtYLMG1-1 of the plant *Arabidopsis thaliana* is exclusively found in the membrane fraction [35]. AtYLMG1-1 also controls nucleoid morphology, whereby knockdown of the gene via expression of an antisense RNA impaired nucleoid partitioning and changed the morphology of nucleoids. Similar results were obtained in the cyanobacterium *Synechococcus elongatus* [35]. Additionally, deletion of *ylmG* in the cyanobacterium *Synechocystis* PCC6803 resulted in thinner cell-wall formation and slower growth rate, though no notable defects in nucleoid segregation was observed [36]. Similar minor effects on cell-wall thickness were observed during cell division in *Streptococcus* [37].

In this work, we show that after the initial localization of SsgB by SsgA at a central location in the hyphae, YlmG plays an important role in the stable localization of SsgB to future septum sites and consequently also in the recruitment of FtsZ. The protein was therefore renamed SepG. The SepG protein localizes to the periphery of the future spore compartments, where it contributes to maintenance of chromosome compaction. In the absence of SepG, the nucleoid expands to the outer limits of the spore compartments, compromising septum synthesis. Taken together, our data provide important new insights into the function of SepG that is well conserved in Gram-positive bacteria, and show that SepG is required for positive control of cell division and has a significant effect on nucleoid morphogenesis in *Streptomyces*.

## 2. Results

### 2.1. Ylmg (SepG) is a predicted membrane protein

To be able to recruit FtsZ, SsgB needs to dock to the membrane, and the lack of a membrane domain suggests that another protein ensures its membrane attachment. In our quest to identify possible partners for SsgB that may coordinate the membrane docking in streptomycetes, we considered the exceptional evolutionary conservation of the FtsZ-recruiting protein SsgB. SsgB is completely conserved in streptomycetes except for aa position 128, a variation which can be used as phylogenetic marker, dissecting the streptomycetes into two different subclades, namely those that sporulate in submerged cultures and those that fail to do so [38]. Conversely, conservation of the SsgB proteins between even related actinomycetes is surprisingly low. We therefore analysed the databases for membrane proteins with similar conservation. Using as criterion more than 90% aa identity between *Streptomyces* species and less than 70% aa identity between orthologues from other actinomycete genera, analysis of the around 1850 putative membrane proteins of *S. coelicolor* was narrowed down to 20 candidate proteins (table 1). Of these, the 94 aa protein YlmG (SCO2078) stood out, with its predicted non-transmembrane domain consisting of aa residues 26–72 showing a very similar conservation as SsgB, with near complete conservation between the *Streptomyces* orthologues (99–100%), but low conservation (50–60% aa identity) between *Streptomyces* and other actinomycetes. Interestingly, the two membrane-spanning domains are less well conserved; these data suggest possible covariation of the central (non-membrane) segment of YlmG and SsgB. Furthermore, *ylmG* lies in the *dcw* cluster, between *sepF* (SCO2079) and *divIVA* (SCO2077). SepF controls the polymerization of FtsZ and, whereas in *Streptomyces* DivIVA controls tip growth [29], it plays a major role in septum-site positioning in *Bacillus subtilis* [8,39]*.* Taken together, the phylogenetic data and genomic location suggest a role for *YlmG* in early cell division events and the possible linkage to *SsgB* was therefore investigated. Considering the genomic location and its potential role in cell division (see below), the protein was renamed SepG.

**Table 1.** Membrane proteins with high conservation in streptomycetes but lower conservation between different actinomycete genera. The 20 best hits are listed.

SCO^a^	protein	function	conservation^b^
SCO1541	SsgB^c^	cell division protein	99
SCO4609	HtpX	metallopeptidase	97
SCO2155	Cox1	cytochrome *c* oxidase subunit I	96
SCO2151	Cox3	cytochrome *c* oxidase subunit III	96
SCO2078	YlmG	membrane protein	96
SCO2944		sugar permease subunit	95
SCO4602	NuoH2	NADH dehydrogenase subunit	95
SCO1389	ClsA	cardiolipin synthase	95
SCO5118	OppB	oligopeptide permease subunit	95
SCO3404	FtsH2^d^	metalloprotease	95
SCO1527		alcohol phosphatidyl transferase	94
SCO2534		hemolysin-like protein	93
SCO1796		stomatin-like protein	93
SCO2150	QcrC	cytochrome *c* heme-binding subunit	93
SCO5670		putative polyamine permease subunit	93
SCO2945		sugar permease subunit	93
SCO2148	QcrB	cytochrome *b* subunit	93
SCO4722	SecY	preprotein translocase subunit	92
SCO1215	CtaG	cytochrome *c* oxidase assembly factor	92
SCO3945	CydA	cytochrome ubiquinol oxidase subunit	92
SCO2087	MurX	phospho-*N*-acetylmuramoyl-pentapeptide-transferase	92

^a^According to the *Streptomyces coelicolor* database numbering.

^b^Average aa identity (in %) between streptomycetes. For this comparison, the eight genomes of the streptomycetes in StrepDB were used. The value is on average 2–3% lower if *S. lividans* is not included.

^c^Included for reference purposes.

^d^Does not occur in all streptomycetes.

The phylogenetic distribution of *sepG* includes the phylum of the actinobacteria (high G+C Gram-positive bacteria), which encompasses both sporulating and non-sporulating genera, several Firmicutes (low G+C Gram-positives), including *Bacillus* and *Streptococcus*, as well as cyanobacteria and chloroplasts. A detailed phylogenetic tree was presented by Kabeya *et al.* [35]. The wide distribution of SepG suggests an ancient common ancestor. Its distant orthologue AtYLMG1-1 from *A. thaliana* was shown to be a membrane protein, and indeed, all prediction programmes strongly suggest that SepG has two membrane-spanning domains at its N- and C-termini, namely from aa 6–25 and from aa 73–90. The different prediction algorithms are uncertain as to the topology of the central domain, but our experiments show that it is most likely to reside inside the cytoplasm (see section Membrane topology of SepG at the end of the Results section).

### 2.2. *sepG* null mutants are compromised in sporulation

To study the function of *sepG* in *Streptomyces*, an in-frame deletion mutant was created in *S. coelicolor* M145. For this, gene replacement construct pGWS731 (see Material and methods section) was introduced into *S. coelicolor* M145 and colonies selected for apramycin resistance (marker for *sepG* disruption) and sensitivity to thiostrepton (marker for the vector). These colonies presumably had *sepG* replaced by the apramycin resistance cassette on the genome. Expression of the Cre recombinase in these cells resulted in a number of markerless deletion mutants, which all had very similar phenotypes with impaired sporulation as judged by the lack of grey pigmentation. After PCR verification of the deletion of *sepG* in these colonies, one representative colony was then selected and designated GAL1.

**Figure 1.**
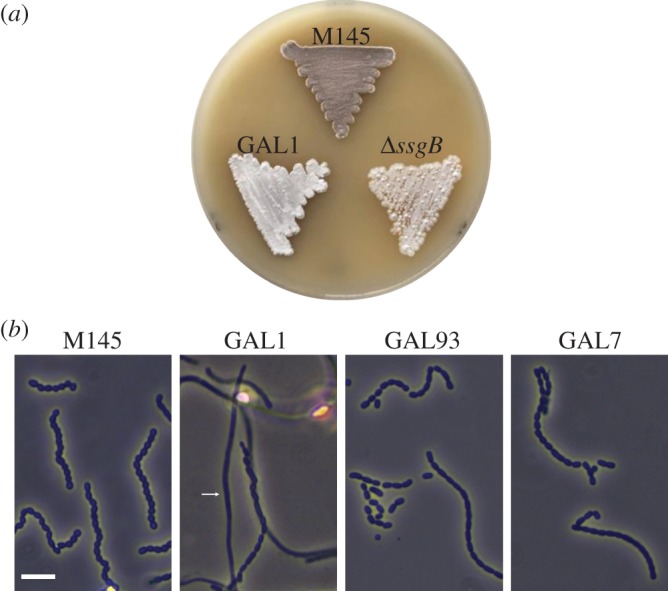
Compromised sporulation of the *sepG* null mutant of *S. coelicolor*. (*a*) Comparison of *S. coelicolor* M145 and its *sepG* null mutant GAL1. Note the lack of grey pigmentation of the *sepG* mutant owing to failure to produce the grey spore pigment. (*b*) Phase-contrast light micrographs of cultures grown on SFM agar against slides. Note that expression of wild-type SepG (GAL93) or SepG–eGFP (GAL7) restores sporulation to the *ylmG* mutant. The arrow indicates a straight aerial hypha in GAL1. All cultures were grown on SFM agar plates for 3 days at 30°C. Scale bar, 5 µm.

Growth on soya flour mannitol (SFM) agar plates showed that *sepG* mutants were impaired in development. While the parental strain *S. coelicolor* M145 developed normal grey-pigmented colonies indicative of sporulation after 3 days of incubation, the *sepG* null mutant GAL1 had a nearly white phenotype, eventually showing light grey pigmentation after elongated incubation (figure 1*a*). The non-sporulating *ssgB* null mutant was used as a reference (figure 1*a*). Sporulation of the strains was investigated by phase-contrast light microscopy of surface-grown colonies after 3 days of growth. The wild-type strain M145 produced abundant spore chains with evenly sized spores, whereas GAL1 produced far fewer spore chains with spores of variable sizes (figure 1*b*). Plasmid pGWS771, a low-copy-number vector expressing *sepG* behind the complete *ftsZ* promoter region (including three promoters and the ribosome binding site sequence), was introduced into strain GAL1 to generate strain GAL93. The restored sporulation of GAL93 indicates that the postponed sporulation was indeed owing to the deletion of *sepG* (figure 1*b*). Strain GAL7, which is GAL1 transformed with SepG localization construct pGWS755, also showed restored sporulation, suggesting that the chimeric SepG–eGFP is functional (figure 1*b*).

**Figure 2.**
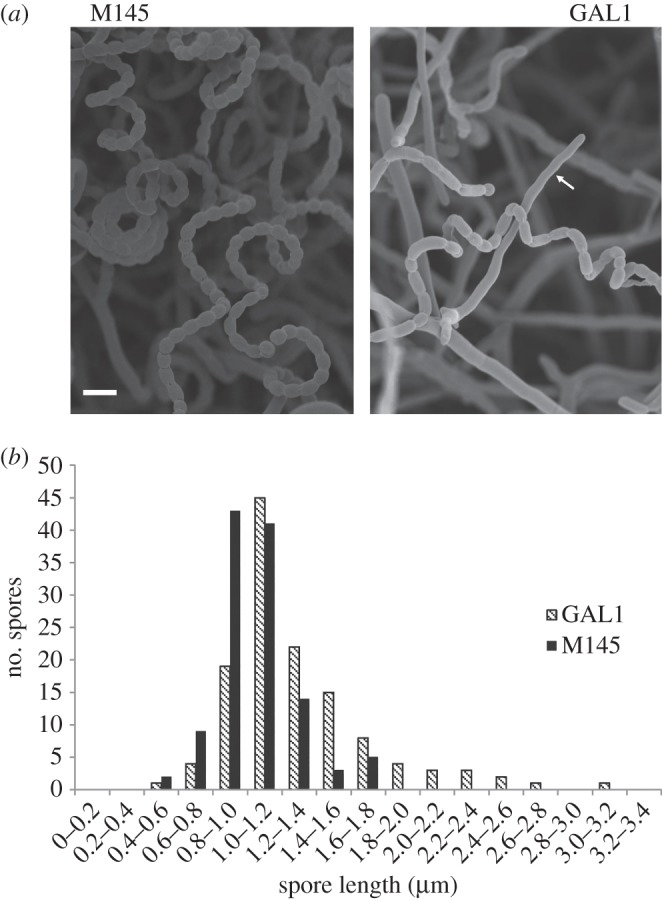
Comparison of spores produced by wild-type *S. coelicolor* M145 and its *sepG* null mutant. (*a*) Cryoscanning electron micrographs of wild-type and *sepG* mutant aerial hyphae. The parental strain sporulated abundantly, while occasional chains of irregularly sized spores were produced by the *sepG* mutant. The arrow indicates a straight aerial hypha in GAL1. Cultures were grown on SFM agar plates for 5 days at 30°C. Scale bar, 2 µm. (*b*) Size distribution of wild-type and *sepG* mutant spores. Spores were measured from a representative number of transmission electron micrographs, so that around 130 spores were measured for each strain. Note that a significant number of *sepG* mutant spores was unusually long.

Closer inspection of the aerial hyphae and spores by cryoscanning electron microscopy (SEM) showed that where the parental strain *S. coelicolor* M145 produced abundant spiralling hyphae and well-developed chains of spores, the *sepG* null mutant predominantly produced straight aerial hyphae, whereby only very few chains of irregularly shaped spores could be identified (figure 2*a*). To quantify the size distribution, approximately 130 spores from the *sepG* mutant and the wild-type strain *S. coelicolor* M145 were measured on high-resolution TEM images (figure 2*b*). While wild-type spores showed the typical Gaussian distribution between 0.8 and 1.2 µm in length with an average size of 0.96 µm, *sepG* mutant spores showed a broader distribution, with an average size of 1.24 µm (*t*-test, *p* < 0.001); in other words, close to 0.3 µm longer on average than wild-type spores. Introduction of plasmid pGWS755 (SepG–eGFP) restored the spore size to 1.06 µm ± 0.05 (*p* > 0.95 compared with the parental strain, *p* < 0.001 compared with the *sepG* mutant). Taken together, these data identify *sepG* as a novel sporulation gene involved in sporulation-specific cell division.

### 2.3. SepG plays a role in spore-wall synthesis and nucleoid morphology

**Figure 3.**
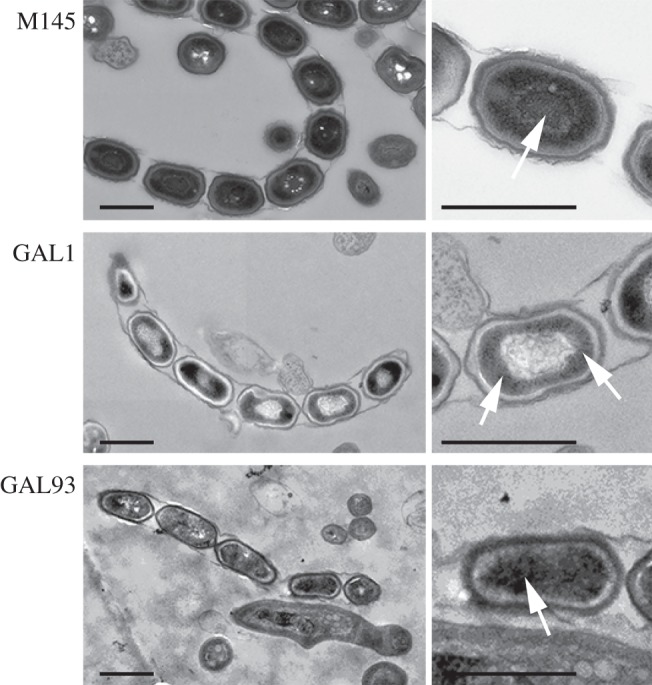
Transmission electron micrographs of wild-type, *sepG* mutant and complemented *sepG* mutant spores. Both a representative overview (left) and close-up (right) are presented. Arrows indicate nucleoids. Note that whereas the wild-type (M145) nucleoid is well condensed and located at the centre of the spores, that of the *sepG* mutant (GAL1) has an unusual distribution. DNA in the *sepG* mutant complemented with wild-type *sepG* (GAL93) was more centrally located than GAL1, although not as well condensed as in wild-type spores. Note the lighter appearance of the spore wall in the *sepG* mutant. Cultures were grown on SFM agar plates for 5 days at 30°C. Scale bar, 1 µm.

To obtain more detailed insights into spore morphology and spore-wall thickness, we performed high-resolution imaging using transmission electron microscopy (TEM) on thin sections. This again revealed the irregular sporulation of the *sepG* mutant. Furthermore, *sepG* mutant spores contained a considerably thinner, less well stained and therefore more electron-lucent cell wall (i.e. lighter in appearance), indicative of altered spore-wall synthesis (figure 3). Another major alteration observed in *sepG* mutant spores was the shape of the spore nucleoid. Rather than a well-condensed nucleoid in the centre of the spores as seen in wild-type spores, nucleoids of the *sepG* mutant spores were less well condensed and primarily localized close to the edge of the spores, with a pattern suggesting an unusual toroidal or doughnut shape (figure 3). Notably, such a toroidal shape is found routinely for nucleoids in developing or germinating spores of *Bacillus* [40]. The DNA was more centrally localized in complemented mutant GAL93, although it was less well condensed than in wild-type spores.

Viability of the *sepG* mutant spores was tested by analysing heat resistance. For this, spores were incubated for 10 min at 60°C and plated onto SFM agar plates. While 45% of the wild-type spores survived the heat treatment, only 8% of the *sepG* mutant spores survived the heat treatment. The defect could be largely complemented by expressing wild-type SepG or SepG–eGFP: spores of GAL7 and GAL93, which are *sepG* mutants harbouring complementation construct pGWS771 and pGWS755, respectively, showed improved heat tolerance, with 32% and 36% of the spores surviving the heat treatment.

**Figure 4.**
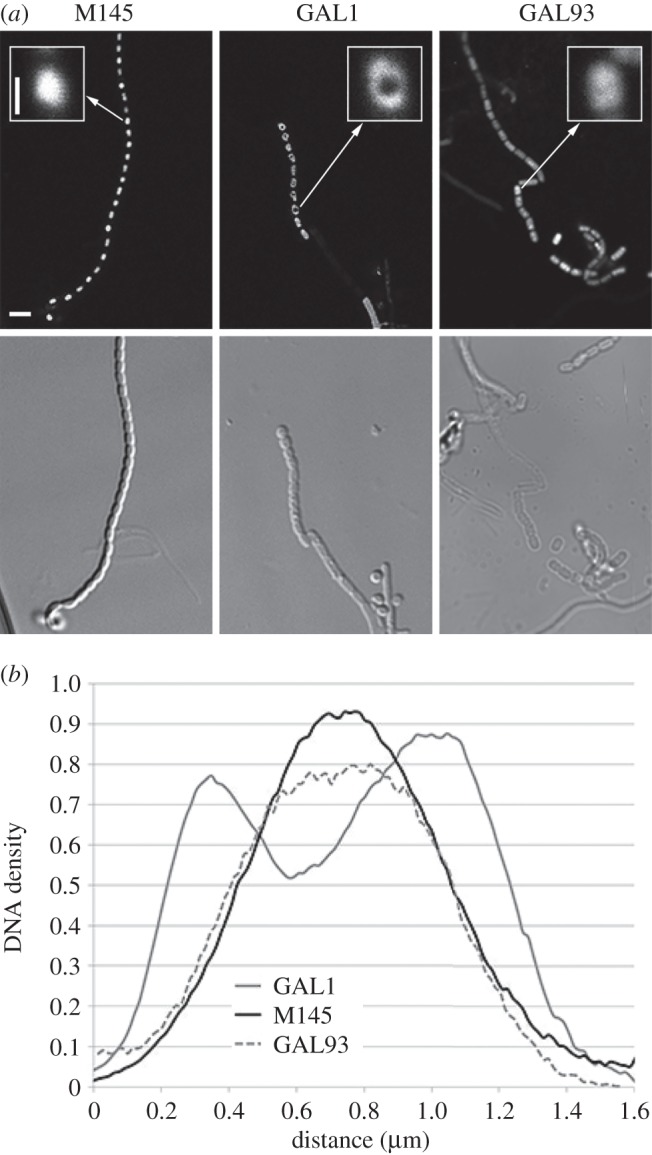
Aberrant nucleoid distribution in *sepG* mutant spores. (*a*) Fluorescence micrographs of nucleoid distribution in the spores; Top, STED images; bottom, light images. DNA staining of wild-type spores (M145, left) shows normal spore lengths and proper nucleoid condensation. In contrast, nucleoids of *sepG* null mutants (GAL1, middle) appear toroid and less well condensed. The aberrant nucleoid shape is complemented by a clone expressing *sepG* (strain GAL93; right), but DNA condensation is still affected. Inset: 7× magnification of representative nucleoids. Cultures were grown on SFM agar plates for 5 days at 30°C. Bar, 2 µm. Bar for inset, 500 nm. (*b*) DNA distribution along single spores in *S. coelicolor* M145, in GAL1 and in GAL93. The *x*-axis represents the distance between two poles of the spores, and the *y*-axis shows the DNA density. Note the double peak for GAL1, highlighting higher density of DNA to the spore periphery when *sepG* is absent, while in wild-type and GAL93 spores the nucleoid is localized to the centre.

To better visualize the nucleoid distribution in wild-type and *sepG* mutant spores, the nucleoids were stained with DAPI and imaged with stimulated emission depletion (STED) microscopy (figure 4*a*). In wild-type spores, the nucleoids were seen as very bright dots that localized to the central part of the spores. Interestingly, in *sepG* null mutants, the nucleoids were less condensed and more dispersed, whereby the centres of the spores were largely devoid of DNA. Spores of strain GAL93, which is the *sepG* null mutant containing plasmid pGWS771 expressing wild-type *sepG*, no longer showed such doughnut-shaped DNA structures, although the nucleoids appeared less well condensed when compared with wild-type spores (figure 4*a*). To provide statistical insights into the frequency of occurrence of this altered nucleoid morphology in GAL1, the percentage of spores with rim-localized DNA in each strain was calculated (using more than 100 spores for each strain). Fourteen per cent of wild-type spores had dents in their intensity profile over a spore, which indicates that the DNA localizes to the spore periphery. For GAL1 that was 74%, while the complemented strain again showed the wild-type proportion of 1 out of 7 (13%) with rim-localized DNA. The average DNA distribution in 10 spores along a spore chain was plotted (figure 4*b*). The wild-type nucleoid profile showed one single peak for the DNA intensity with regular height and width, suggesting well-condensed DNA. Conversely, nucleoid profiles of GAL1 displayed a clear intensity drop in the middle of the spore, indicating that the DNA in each spore compartment localizes more towards the edge of the spores and away from the centre. DNA distribution in the complemented mutant GAL93 was largely restored, showing a single peak in the middle of the spores.

Thus, TEM, STED and nucleoid distribution data strongly suggest that SepG is required for proper nucleoid compaction during sporulation. It is as yet unclear if this is a direct or indirect effect of the deletion of *sepG.* However, the defect can be complemented by introduction of wild-type copy of *sepG*, underlining that the absence of SepG is the primary cause of the defect.

### 2.4. Localization of SepG

To determine the localization of SepG in *S. coelicolor*, C-terminal fusions with either enhanced green fluorescent protein (eGFP) or (monomeric) mCherry were expressed from the *ftsZ* promoter region (from plasmids pGWS755 and pGWS791, respectively). Preliminary transcript analysis and DNA sequence analysis failed to identify promoter sequences immediately upstream of *sepG*, whereas promoter activity was identified inside of the upstream-located *sepF* (data not illustrated). To avoid including *sepF* sequences, we instead directed transcription of *sepG* from the promoter region of *ftsZ*, so as to coordinate transcription with cell division. The *ftsZ* promoter region consists of three promoters, one of which is expressed primarily during vegetative growth, one during sporulation and one is constitutive [41]. We will further refer to this as the *ftsZ* promoter region. Various independent DNA microarray analyses from our laboratory indicate that *sepG* and *ftsZ* have a similar expression profile throughout growth, whereby *sepG* is consistently expressed about 2–2.5 times higher than *ftsZ* in *S. coelicolor* M145 [42]; this implies that use of the *ftsZ* promoter region will not lead to overexpression of *sepG*, nor with low-copy-number vectors. Indeed, introduction of pGWS755 or pGWS791 restored sporulation to the *sepG* null mutant, showing that both SepG fusion proteins are functional and expressed properly *in vivo*. SepG–mCherry and SepG–eGFP had the same localization profile, providing further support for the functionality and reproducibility of the localization experiments (see also below).

**Figure 5.**
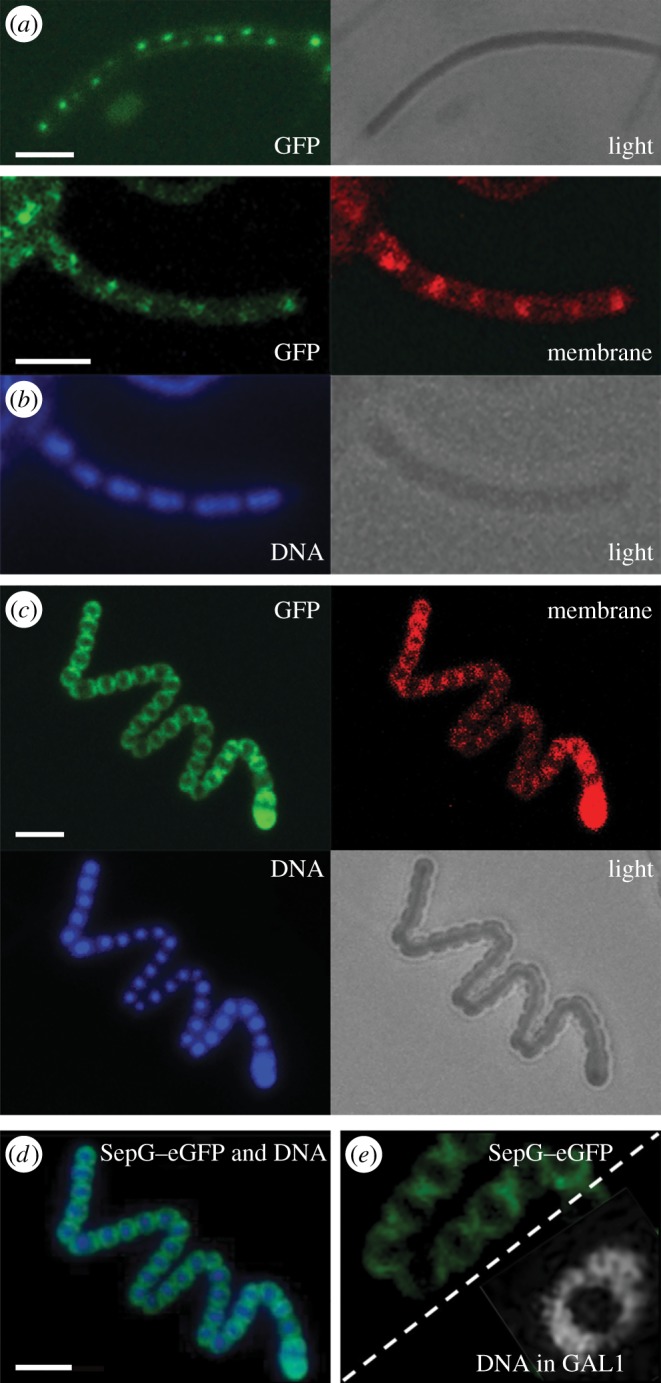
Localization of SepG in *S. coelicolor*. (*a*–*c*) Sporogenic aerial hyphae of *S. coelicolor* M145 were imaged by fluorescence microscopy visualizing SepG–eGFP, membrane (stained with FM5–95), DNA (stained with DAPI) and a light micrograph at the onset of sporulation in aerial hyphae (*a*), during sporulation (*b*) and spore maturation (*c*). (*d*) A merged image of SepG–eGFP and the DNA taken from (*c*). (*e*) SepG–eGFP in wild-type cells (strain GAL7; left) showing a similar localization in the spores as the DNA in the *sepG* null mutant (strain GAL93; right), suggesting that SepG aids in DNA condensation in the centre of the spores. Note that the images in panel (*e*) are only presented for comparison, as the sepG–GFP data were obtained from panel (*d*) (FM imaging), while the nucleoid was obtained from figure 4*a* (STED imaging). Scale bar, 3 µm.

**Figure 6.**
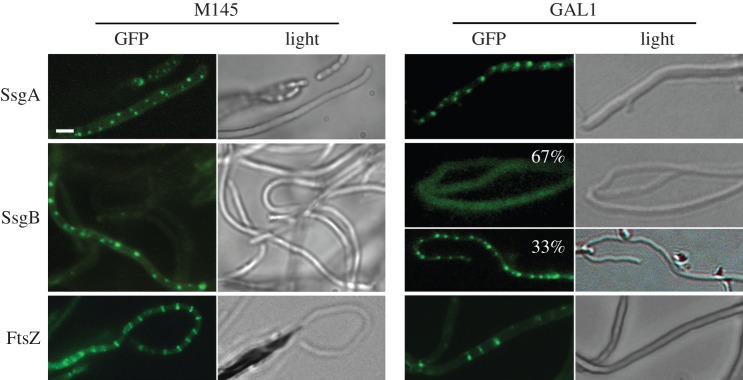
Effect of SepG on the localization of eGFP fusions of SsgA, SsgB and FtsZ. All proteins localize correctly in wild-type cells (M145, left). In contrast, SepG is required for the formation of ladders of FtsZ or for the localization of SsgB at division sites. SsgA has a normal focal pattern in the *sepG* mutant, whereas focal patterns for SsgB were seen in around 33% of the aerial hyphae (67% of the GAL1 aerial hyphae showed diffuse localization of SsgB); SsgB fails to localize to the future septum sites in sporogenic aerial hyphae of GAL1. The occasional Z rings formed in *sepG* null mutant GAL1 are also seen in *ssgB* mutants [27]. Cultures were grown on SFM agar plates for 5 days at 30°C. Scale bar, 1 µm (for all images).

Confocal fluorescence microscopy was applied to investigate SepG–eGFP localization in wild-type cells and the *sepG* mutant. The pattern of SepG–eGFP localization was dynamic and developmental stage-dependent. In young aerial hyphae, when the chromosomal DNA was still uncondensed and septal membrane synthesis had not yet initiated, SepG–eGFP formed distinct and widely spaced foci (figure 5*a*). Such a localization pattern is very similar to that of the initial stage of SsgB localization, prior to its septum-site localization [27] (figure 6). As development progressed, nucleoids segregated and septal ladders became apparent, whereas SepG–eGFP disappeared (figure 5*b*). During spore maturation the nucleoids condensed, and at this stage, SepG–eGFP relocalized to the sites of active cell-wall remodelling (figure 5*c*), with a ring of SepG surrounding the nucleoid (figure 5*d*). The fluorescence images strongly suggest that in *sepG* mutant spores, the nucleoid takes up the space normally occluded by a SepG-dependent mechanism (figure 5*e*). In mature spores, which are physically separated, no GFP signal could be observed, suggesting that SepG eventually disappears (not illustrated).

### 2.5. Localization of SsgB to septum sites depends on SepG

We previously showed that FtsZ is directly recruited by SsgB in an SsgA-dependent manner, followed by FtsZ-dependent recruitment of the other divisome components [27]. The phenotype of the *sepG* null mutants prompted us to investigate whether the localization of one or more of these proteins was affected by the absence of SepG. To address this question, the integrative vectors pGWS116, pGWS526 or pKF41, expressing SsgA–eGFP, SsgB–eGFP or FtsZ–eGFP, respectively, were introduced into the *sepG* null mutant. While SsgA–eGFP localized normally in the mutant, SsgB–eGFP showed two patterns of localization, namely diffuse along the hyphal periphery or foci at what are most likely the future septum sites. The diffuse pattern was mainly observed in *sepG* null mutants, whereas foci were predominantly seen in the parental strain M145. In line with the delocalized pattern of the FtsZ-recruiting SsgB protein, the *sepG* mutant also failed to produce the typical ladder-like patterns of FtsZ–eGFP seen in wild-type hyphae (figure 6). Only occasional septa were formed, a phenotype that is also observed in *ssgB* null mutants and other sporulation (*whi*) mutants [27].

SsgB–eGFP still formed distinct foci, but mainly in the middle of young aerial hyphae (which can be distinguished from pre-sporulating aerial hyphae as they are narrower in width [18]), representing the first stage of SsgB localization (figure 6). However, in contrast to wild-type cells [27], in *sepG* null mutants the SsgB foci subsequently disappeared (figure 6), indicating that the stable localization of SsgB requires SepG. In wild-type cells, SsgB–eGFP foci appeared in 80% of the hyphae with around 1.25 ± 0.14 µm spacing between them. While in *ssgA* and *sepG* null mutants, SsgB–eGFP foci were only apparent in 30% and 33% of the hyphae, spaced by 0.53 ± 0.07 and 0.57 ± 0.04 µm, respectively. Thus, after the SsgA-mediated initial localization of SsgB, SepG directly or indirectly acts to ensure the localization of SsgB at future division sites at the membrane, which is a crucial step in initiating sporulation-specific cell division. This is consistent with SepG being required for the septum-site localization of SsgB after its initial localization by SsgA (figure 6).

This raised the question whether SepG directly interacts with SsgB. To investigate this *in vivo*, we applied Förster resonance energy transfer (FRET), which allows the identification of direct molecular interactions. As low-wavelength fluorophore, eGFP was used, whereas mCherry was used as longer-wavelength fluorophore. SepG–mCherry was introduced into *S. coelicolor* M145 expressing either SsgA–eGFP or SsgB–eGFP to generate strains GAL94 and GAL95, respectively; SepG–eGFP was introduced into *S. coelicolor* M145 expressing FtsZ–mCherry to generate strain GAL96. SepG–eGFP was also introduced in M145 expressing free mCherry under the control of the *gap1* promoter (from SCO1947, the gene for glyceraldehyde-3-phosphate dehydrogenase) to generate strain GAL103. Strain GAL102, which is *S. coelicolor* M145 co-expressing SsgB–eGFP with FtsZ–mCherry, was used as positive control, considering the direct interaction between SsgB and FtsZ [27]. As negative control we used *S. coelicolor* K202, which expresses FtsZ–eGFP alone.

**Figure 7.**
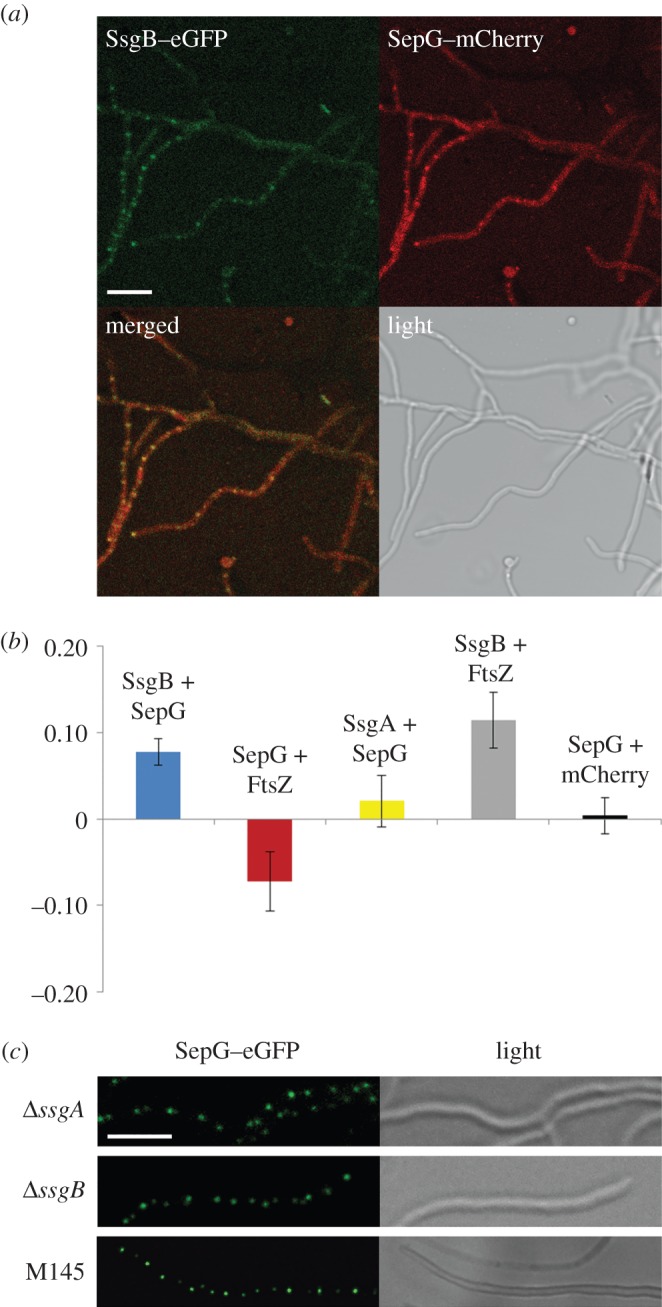
FRET imaging of the interaction between SepG and SsgB. (*a*) FM images showing the colocalization of SsgB and SepG. Scale bar, 5 µm. (*b*) Graphical representations of the FRET data. All efficiencies are represented relative to the control (which was set to zero). FRET data are summarized in table 2. (*c*) FM images showing localization of SepG–eGFP in *ssgA*- and *ssgB-*null mutants. Scale bar, 2 µm.

The data are summarized graphically in figure 7*b*, whereby the negative control was set to zero. GAL102 showed a FRET efficiency consistent with the short intermolecular distance between SsgB and FtsZ 1.08 ± 0.03 (table 2; *p* < 0.01 compared with SepG-free mCherry). The negative control FtsZ–eGFP showed a similar fluorescence intensity before and after acceptor photobleaching (intensity before bleach is set to 1) of 0.97 ± 0.02, consistent with a lack of interaction with an acceptor (table 2). Importantly, SepG and SsgB colocalized, as shown by the more than 90% overlap of their foci in approximately 100 hyphae (figure 7*a*), and the FRET data strongly suggest close interaction between them, with an increase in fluorescence intensity to 1.05 ± 0.02 (*p* < 0.01), an intermolecular distance close to that between the known partners SsgB and FtsZ. No interaction was seen between SepG and SsgA (no fluorescence intensity increase; 0.99 ± 0.03, *p* > 0.4), between SepG and FtsZ (0.90 ± 0.03, *p* > 0.1) or between SepG and free mCherry (0.97 ± 0.02).

**Table 2.** FRET efficiencies for different combinations of donor (eGFP) and acceptor (mCherry).

	GAL95	GAL96	GAL94	GAL102	GAL103	K202
strain	SsgB + SepG	SepG + FtsZ	SsgA + SepG	SsgB + FtsZ	SepG + mcherry	FtsZ
average	1.05	0.90	0.99	1.08	0.97	0.97
standard deviation	0.02	0.03	0.03	0.03	0.02	0.02
*n*	24	18	11	6	12	9

We then wondered if SepG could localize independent of SsgA or SsgB, which should be the case if SsgB localization depends on SepG but not vice versa. To test this, construct pGWS756, expressing SepG–eGFP, was introduced into the *ssgA* and *ssgB* null mutants. SepG formed distinct and widely spaced foci in the aerial hyphae in both *ssgA* and *ssgB* null mutants, with a pattern very similar to that in wild-type cells (figure 7*c*). SepG–eGFP also localized at the intersection of prespores in wild-type cells (figure 5); because *ssgA* and *ssgB* mutants do not produce spores, SepG–eGFP was only visualized as foci in these mutants.

Taken together, our data show that the stable localization of the FtsZ-recruiting SsgB requires SepG, and not vice versa. FRET experiments suggest that SepG and SsgB directly interact, whereas the lack of interaction of SepG with SsgA or FtsZ is consistent with the relocation of SepG prior to assembly of the other divisome members. We also sought to confirm this by two-hybrid experiments using the central (cytoplasmic) domain of SepG as the bait, but—like for many other cell division proteins—this failed to provide conclusive data, both in direct studies with SepG and SsgB, or in a library screen with (partial) SepG as the bait (now shown). The precise way that SsgB and SepG interact is under investigation.

### 2.6. Membrane topology of SepG

All membrane prediction programs (e.g. DAS, TMPred) predict with high probability scores that SepG is a membrane protein with two TM domains. To establish if indeed SepG is integrated in the membrane, and the SepG–eGFP fusion protein is stable, we first estimated the mobility of SepG–eGFP using fluorescence recovery after photobleaching (FRAP). Freely mobile fusion proteins would have a half time of recovery of around 0.03 s in the cytoplasm, whereas for membrane-associated fusions, the recovery should be much slower (in the order of seconds) [43]. In *Streptomyces*, the half time of recovery for freely mobile eGFP with *egfp* transcribed from *gap* promoter proteins was around 0.8 s, whereas for SepG–eGFP it was 42.3 ± 3.3 s. TatA–eGFP was included as a positive control for a membrane protein, and showed a half time of recovery of 82.3 ± 11.5 s. These data clearly show that SepG–eGFP recovers much slower than free eGFP proteins, consistent with SepG–eGFP localized inside the membrane (and no evidence of free eGFP such as after inadvertent cleavage of the SepG–eGFP fusion protein *in vivo*).

To obtain further experimental evidence that SepG is a membrane protein and to establish whether the central domain is localized inside the cytoplasm or outside the cell, we used the β-lactamase reporter system for transmembrane proteins, an elegant system developed originally by Broome-Smith *et al.* [44] that allows establishing the orientation of transmembrane (TM) domains. BlaM is a β-lactamase that is very efficiently translocated over the membrane, and if it is fused behind a TM domain with an inside–outside orientation, it will result in BlaM translocation over the membrane and thus convey ampicillin resistance to the cells [44–46]. Construct pGWS755, which expresses SepG–eGFP, was used as the basis for further derivatives. While the construct is made for expression in *Streptomyces*, introduction of pGWS755 in *E. coli* JM109 also results in the production of SepG–eGFP, as shown by fluorescence imaging. This indicates that at least one of the *S. coelicolor ftsZ* promoters is actively transcribed in *E. coli*. Interestingly, the fluorescent fusion protein localized specifically to the poles of *E. coli* cells (data not illustrated). Derivatives with various parts of *sepG* fused in front of *blaM* were made as described in the Material and methods section. These allow the expression in *E. coli* of the following fusion products: SepG-BlaM (to probe full-length SepG; pGWS793), SepG_1–40_-BlaM (TM domain 1; pGWS794) and SepG_41–94_-BlaM (TM domain 2; pGWS795). In constructs pGWS793, pGWS794 and pGWS795, only the part of *blaM* encoding the mature protein (starting with HPETLVK) was used. As a positive control BlaM including its signal peptide was expressed from construct pGWS796 under the control of the *ftsZ* promoter region. Low-copy-number plasmid pHJL401 (which carries *blaM* as resistance marker) was also used as a positive control.

**Figure 8.**
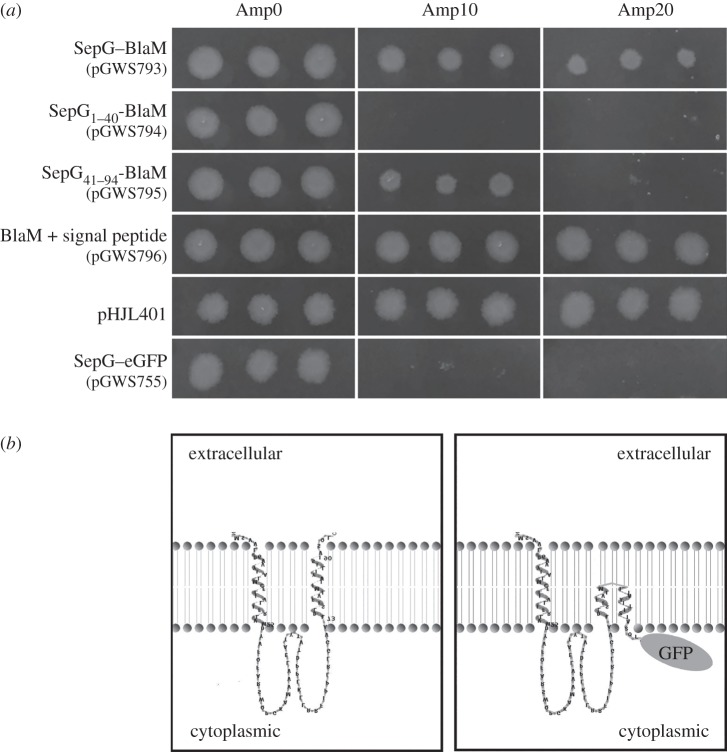
Membrane topology of SepG. (*a*) Growth of *E. coli* JM109 carrying plasmids expressing different BlaM fusions, namely full-length SepG–BlaM (pGWS793); N-terminal SepG_1-40_-BlaM (pGWS794; first TM domain of SepG); C-terminal SepG_41–94_-BlaM (pGWS795; second TM domain of SepG); BlaM with signal sequence expressed from the *ftsZ* promoter(s) (pGWS796; positive control); BlaM with signal sequence expressed from its native promoter (pHJL401; positive control); and pGWS755 expressing SepG–eGFP (pGWS755; negative control). Transformants were grown overnight at 37°C on LB agar plates with 0, 10 or 20 µg ml^−1^ ampicillin. (*b*) Topological model of SepG. Left, wild-type SepG, with the topology model generated using TMRPRES2D [47]. Right, model of SepG fused to eGFP, drawn based on the wild-type SepG model.

Constructs were introduced into *E. coli* JM109 and transformants tested for their ability to grow on ampicillin (see Material and methods). All transformants grew equally well on LB agar plates without ampicillin (figure 8*a*). The negative control (JM109 harbouring pGWS755 expressing SepG–eGFP) failed to grow on plates containing ampicillin, whereas the positive controls, JM109 with either plasmid pHJL401 or with a construct expressing BlaM with signal sequence from the *S. coelicolor ftsZ* promoter region (pGWS796), grew well under all conditions (figure 8*a*). This again shows that at least one of the *S. coelicolor ftsZ* promoters results in active transcription in *E. coli*. *E. coli* cells expressing BlaM fused behind the N-terminal part of SepG (i.e*.* the first TM domain, pGWS794) failed to grow on ampicillin, whereas constructs where BlaM was fused behind full-length SepG (pGWS793) or the C-terminal part of SepG (containing only the second TM domain; pGWS795) conveyed ampicillin resistance, albeit with smaller colonies in the case of pGWS795 (figure 8*a*). These results strongly suggest that the N-terminal domain of SepG is oriented from outside to inside the cell, whereas the C-terminal domain is oriented towards the outside of the cell. At higher concentrations of ampicillin (20 µg ml^−1^), *E. coli* cells expressing SepG–BlaM did grow, but those containing only the C-terminal TM domain in front of SepG–BlaM failed to grow, perhaps because only a smaller fraction of the proteins is correctly oriented in the membrane when a single TM domain is present.

Growth of all transformants was then analysed in liquid LB with or without ampicillin, using a Bioscreen C automated microwell plate reader with continuous shaking and temperature control (three independent colonies and three replicates per colony). These data were fully consistent with the observations made when the transformants were grown on LB agar plates. *E. coli* expressing BlaM fused behind full-length SepG grew very well at all ampicillin concentrations, as did transformants harbouring the positive control plasmids pGWS796 and pHJL401, which express *blaM* from the *S. coelicolor ftsZ*p and from the native *blaM*p, respectively (electronic supplementary material, figure S1). Conversely, *E. coli* cells expressing N-terminal SepG_1–40_-BlaM or SepG–eGFP failed to grow in the presence of ampicillin. Transformants expressing BlaM fused behind the C-terminal part SepG_41–94_ did grow at 10 µg ml^−1^ ampicillin, but only after prolonged incubation, demonstrating that a single TM domain is not sufficient to provide stable integration of proteins in the membrane (electronic supplementary material, figure S1).

## 3. Discussion

The control of cell division in the multicellular streptomycetes is very different from that in for example *E. coli* and *B. subtilis*. During sporulation, the aerial hyphae differentiate into long chains of spores, and the more or less simultaneous formation of many septa results in spectacular ladders of Z rings [17]. The canonical control systems and septum-localizing proteins (Min, Noc, FtsA, ZipA, ZapA, EzrA, etc.) found in bacteria that divide by binary fission are all absent in streptomycetes, and instead actinomycete-specific proteins control the localization of the septa. In streptomycetes, cell division is positively controlled, whereby FtsZ is actively recruited to septum sites by the action of the actinomycete-specific SsgB [27].

In this work, we show that *ylmG* (*sepG*), which lies between *sepF* and *divIVA*, and is one of the last genes in the *dcw* cluster whose function had not yet been established, plays a major role in the control of sporulation-specific cell division in *S. coelicolor*. SepG is a predicted membrane protein, and the distant SepG orthologue AtYLMG1-1 of *A. thaliana* is exclusively found in membrane fractions [35]. Our data show that this is also the case in *S. coelicolor* (see below). In *Streptomyces*, SepG helps to orchestrate the earliest stage of sporulation-specific cell division, as it is required for the correct localization of SsgB to future septum sites. After the initial localization of SsgB by SsgA to a central position in the aerial hyphae, this is the next crucial step in the positive control of cell division in *Streptomyces*, followed by the recruitment of FtsZ by SsgB. In addition, at a later stage of spore development, SepG helps maintaining nucleoid shape, either directly or indirectly. In wild-type cells, SepG localizes in a ring at the periphery of the spores and surrounding the nucleoid, whereas in *sepG* null mutants, large doughnut-shaped (toroid) nucleoids are formed accompanied by thinner spore walls, suggesting imperfect cell-wall synthesis, which is consistent with the observed reduced heat resistance of the spores. One explanation is that the expanded nucleoids in spores of *sepG* null mutants enforce the formation of larger spores, and hence with thinner cell walls assuming that the same amount of cell-wall material is available per spore. In *A. thaliana*, the SepG orthologue AtYLMG1-1 also plays a role in controlling nucleoid morphology, whereby knockdown of the gene impaired nucleoid partitioning and changed the shape of the nucleoid. Similar results were obtained in the cyanobacterium *S. elongatus* [35]. This therefore is most likely to be an inherent feature of SepG that remained conserved through evolution. After the localization of SsgB to the future sites of division, SepG continues to follow the spore-wall synthetic machinery, and ensures the coordination of spore-wall synthesis with nucleoid compaction by a mechanism that as yet remains unresolved. In line with its more prominent role during sporulation-specific cell division, previous microarray data showed that, as for *ssgB*, the transcription of *sepG* is enhanced at the onset of sporulation [42,48].

The fact that SepG localizes normally in *ssgA* and *ssgB* mutants, whereas conversely, SsgB fails to localize properly in *sepG* mutants, supports a model wherein SepG ensures the correct localization of the FtsZ-recruiting SsgB protein. The failure of SsgB to find the septum sites thereby explains the mislocalization of FtsZ in *sepG* null mutants. As an alternative to recruitment, SepG may also be involved in stabilizing SsgB complexes in the sporogenic hyphae. A question to answer is whether SepG directly interacts with SsgB. FRET data provide *in vivo* evidence that supports a direct interaction between SepG and SsgB during the onset of cell division. In further support of this notion, specific FRET signals were only obtained when SepG and SsgB were analysed, but not for SepG with SsgA, FtsZ or free mCherry. Interestingly, the central domain of SepG shows a similar conservation as SsgB, with extremely high conservation in streptomycetes, but low conservation between different actinomycete genera. Such a conservation pattern is not evident in the predicted TM domains, and the suggestive covariation in terms of aa conservation in different bacteria between the SepG cytoplasmic domain and SsgB provides supporting phylogenetic evidence consistent with possible interaction between SsgB and SepG. No other interaction partners were identified in a library screen with SepG as the bait. Furthermore, no interaction was observed between SepG and SsgA. The way in which SepG and SsgB interact is currently under investigation in our laboratory.

Thus, in terms of the control of cell division, our data are consistent with a model wherein SepG recruits SsgB to the future septum sites, and SsgB subsequently recruits FtsZ. While SsgB and FtsZ remain together a divisome protein, SepG does not become part of the divisome, but instead moves away from SsgB, whereby our data suggest that it may subsequently relocate to the cell-wall synthetic machinery. While relatively little is known of nucleoid dynamics in *Streptomyces*, we can try to glean information from the well-studied *B. subtilis*, although *Bacillus sepG* null mutants have no apparent phenotype [8]. During vegetative growth the nucleoid localizes close to mid-cell in *B. subtilis*, but its shape changes during the onset of sporulation, and eventually a doughnut-shaped ring is formed similar to that found in *S. coelicolor sepG* mutants, with a diameter of around 1 µm [40]. During germination, these rings disappear and the nucleoids take up the diffuse lobular shape typical of vegetative cells [49]. The expansion of the nucleoid towards the spore periphery probably disturbs the cell cycle, which may trigger a nucleoid-occlusion-like mechanism to prevent septum synthesis close to the nucleoid; however, such a mechanism has not yet been identified in streptomycetes, neither in wild-type cells nor in mutants disturbed in DNA partitioning, such as *parA* and *parB* mutants of *S. coelicolor*.

An important question to resolve was what the membrane topology is of SepG, and in particular whether the small central (soluble) region is oriented towards the inside or the outside of the cell. The slow FRAP recovery, with a half time of recovery in the order of a minute, is consistent with SepG being a membrane protein, as freely diffusible GFP/mCherry would have a half time in the millisecond range. For the localization, studies both SepG–GFP and SepG–mCherry fusion proteins were used, and either protein is functional, because they could restore sporulation to the *sepG* null mutant. Furthermore, our FRET data on the C-terminal fusion proteins (either mCherry or GFP derivatives) show that the fluorophore is located inside the cytoplasm, and that most if not all of the fusion proteins in the cell are intact. Membrane topology analysis based on the BlaM reporter system [44] showed that the central part of SepG is located inside the cell. The first TM domain was able to direct BlaM to the cytoplasm, whereas fusion behind full-length SepG or behind the second TM domain directed BlaM to the periplasm, strongly suggesting that the first TM domain of SepG is oriented from outside to inside, whereas the second TM domain is oriented from inside to outside. A topological model for SepG is presented in figure 8*b*, including a suggested topology for the SepG–eGFP fusion protein. The orientation of the central domain towards the inside of the cell is consistent with a model wherein SepG interacts with SsgB and then recruits SsgB to the membrane. In the chimeric SepG–eGFP protein, eGFP will probably prevent full membrane spanning of the C-terminal TM domain of SepG, which may also explain why the fusion protein can only partially restore sporulation to *sepG* mutants.

Finally, the principles discovered in this work for *Streptomyces* may well translate to lower Gram-positives such as *Bacillus* and *Streptococcus*. This is among others based on strong phylogenetic evidence, because the location of *sepG* between *divIVA* and *sepF* was maintained during several hundred millions years of evolution, whereby *ylmH* was lost in actinomycetes. This suggests functional overlap between the *Bacillus* and *Streptomyces* orthologues, despite the apparent absence of SsgB in firmicutes. A key observation may well be that, as in *Streptomyces*, a cell-division control mechanism that is independent of the negative control systems Min and Noc has recently been identified in *B. subtilis*. It was shown that the Z ring can be positioned at division sites in the absence of Min and Noc, and that in Noc^−^ cells the Z rings have a preference for the mid-cell position between the two nucleoids [32]. Conceivably, therefore, SepG may have a similar role in division-site selection in *B. subtilis* as in *Streptomyces*, but such a role would only be obvious if *sepG* were to be studied—and, if at all possible, mutated—in the absence of Noc. Further molecular insights into the function of SepG in streptomycetes will shed more light on the way the positive control of cell division is governed in these multicellular bacteria, as well as on the yet poorly understood mechanisms that prevent nucleoid damage during septum synthesis and cell-wall remodelling.

## 4. Material and methods

### 4.1. Bacterial strains and media

The bacterial strains used in this work are listed in electronic supplementary material, table S1. *E. coli* strains JM109 [50] and ET12567 [51] were used for routine cloning and for isolation of non-methylated DNA, respectively. *E. coli* transformants were selected on LB agar media containing the relevant antibiotics and grown O/N at 37°C. *S. coelicolor* A3(2) M145 [52] was used as parental strain to construct mutants. The *ssgA* null mutant GSA3 [53] and *ssgB* null mutant GSB1 [54] of *S. coelicolor* M145 were obtained from the Leiden University strain collection. All media and routine *Streptomyces* techniques are described in the *Streptomyces* manual [52]. Yeast extract-malt extract and tryptone soy broth with 10% sucrose were the liquid media for standard cultivation. Regeneration agar with yeast extract was used for regeneration of protoplasts and with appropriate antibiotics for selection of recombinants [52]. SFM agar plates were used to grow *Streptomyces* strains for preparing spore suspensions and for morphological characterization and microscopy.

### 4.2. Plasmids and constructs

All plasmids and constructs described in this work are listed in electronic supplementary material, table S2 and oligonucleotides in electronic supplementary material, table S3. PCRs were carried out with Phusion enzyme (Finnzymes, Bioké, Leiden, The Netherlands) as previously described [55].

#### 4.2.1. Constructs for gene disruption

The strategy for creating knock-out mutants is based on the unstable multi-copy vector pWHM3 [56], essentially as described previously [48]. The −1442/+6 and +277/+1521 regions relative to the translational start of *ylmG* (*sepG*; SCO2078; 285 nt total) were amplified by PCR from the *S. coelicolor* M145 genome using primer pairs ylmG_LF_1442 and ylmG_LR + 6, and ylmG_RF + 277 and ylmG_RR + 1521, respectively (electronic supplementary material, table S3). Fragments were then cloned into *Eco*RI/*Bam*HI-digested pGWS725, with the oligonucleotides designed such as to create a unique *Xba*I site in-between the flanking regions. The apramycin resistance cassette *aac(3)IV* flanked by *loxP* sites was then cloned via the engineered *Xba*I site to generate knock-out construct pGWS731. The presence of *loxP* sites allows the efficient removal of the apramycin resistance cassette from the chromosome following the introduction of plasmid pUWLCRE that expresses the Cre recombinase [57]. For complementation of the *sepG* null mutant the coding sequence (+1/+294 region) of *sepG* under control of the *ftsZ* promoters (pGWS771) was inserted into the integrative vector pSET152 [58], which integrates at the attachment site for bacteriophage ΦC31 on the *S. coelicolor* genome.

#### 4.2.2. Constructs for enhanced green fluorescent protein and mCherry fusion proteins

To obtain a construct expressing SepG–eGFP, we used plasmid pKF41, which expresses FtsZ–eGFP from the native *ftsZ* promoter region [59]. The insert was excised using restriction enzymes *Bgl*II and *Not*I, cloned into the integrative vector pSET152. To replace the *ftsZ* coding region by that of *sepG*, the construct was digested with *Stu*I and *Bam*HI, and the *sepG* gene was PCR-amplified from the genome of *S. coelicolor* using primer pair ylmG_F + 1 and ylmG_R + 282. The PCR product was subsequently cloned as a *Stu*I–*Bam*HI fragment in between *ftsZ*p and *egfp* to generate pGWS755. The insert of pGWS755 was then moved as an *Eco*RI–*Xba*I fragment into the low-copy-number vector pHJL401 [60] to generate pGWS756. Vector pHJL401 is a stable vector with (very) low-copy-number (1–5 copies per chromosome), which is very well suited for complementation studies [61]. To generate similar constructs expressing SepG–mCherry, the *egfp* gene in pGWS755 was removed by digestion with *Bam*HI and *Not*I and replaced by the gene for mCherry to generate pGWS791.

#### 4.2.3. Constructs for β-lactamase experiments

Various constructs were created to study the transmembrane topology of SepG in *E. coli* using BlaM (β-lactamase) as a reporter, according to a system that was described previously [44]. The gene for eGFP in pGWS755 was replaced by the *blaM* gene without the sequence encoding the signal peptide (i.e. from HPETLVK to the end) to generate construct pWGS793. The *blaM* gene was amplified by PCR from pHJL401 using primers BlaM_F_EB and BlaMR_XH, adding 36 nt encoding a 12 amino acid linker in primer BlaM_EB as described [44]. The resulting construct pGWS793 expresses SepG-BlaM hybrid protein from the *ftsZ* promoter region. The part of *sepG* encoding the N-terminal 40 aa including the first TM domain was amplified from *S. coelicolor* M145 genomic DNA using primers YlmG_F + 1 and YlmG_R + 120. The fragment was cloned into pGWS793 as a *Stu*I–*Bam*HI fragment to replace full-length *sepG*, resulting in pGWS794. Similarly, the part of *sepG* encoding aa 41–94 of SepG including the second TM domain was amplified from *S. coelicolor* M145 genomic DNA using primers YlmG_F + 121 and YlmG_R + 282 and cloned into pGWS793, to replace full-length *sepG*, resulting in pGWS795. Full-length *blaM* (including the sequence encoding the signal sequence for secretion) was amplified by PCR from pHJL401 using primers BlaM_F + 1_ES and BlaM_R_XH. This DNA fragment was cloned into pGWS793 as a *Stu*I–*Xba*I fragment so as to replace *sepG*-*blaM*, resulting in construct pGWS796, which expresses full-length BlaM.

### 4.3. Microscopy

Sterile coverslips were inserted at an angle of 45° into SFM agar plates, and spores of *S. coelicolor* and derivatives were carefully inoculated at the intersection angle. After incubation at 30°C for 3–5 days, coverslips were positioned on a microscope slide pre-wetted with 5 µl of 1× PBS. Fluorescence and corresponding light micrographs were obtained with a Zeiss Axioscope A1 upright fluorescence microscope (with an Axiocam Mrc5 camera at a resolution of 37.5 nm pixel^−1^). The green fluorescent images were created using 470/40 nm band pass excitation and 525/50 band pass detection, whereas for the red channel 550/25 nm band pass excitation and 625/70 nm band pass detection was used [62]. DAPI was detected using 370/40 nm excitation with 445/50 nm emission band filter. For membrane staining, FM5–95 was used and for DNA staining DAPI (all obtained from Molecular Probes). To obtain a sufficiently dark background, all images were corrected by setting the signal outside the hyphae to zero. These corrections were made using Adobe Photoshop CS4.

Chromosome distribution in spores of *S. coelicolor* strains was studied using stimulated emission depletion (STED) microscopy. For this, patches of the different *Streptomyces* strains were grown on SFM agar plates and incubated at 30°C for 5 days. Live cells were stained with Syto 9 (0.5 mM) for 5 min, after which STED was performed with a Leica TCS STED CW system with 488 nm excitation (5% laser power) and depletion at 592 nm (30% laser power).

Morphological studies on surface-grown aerial hyphae and/or spores by SEM were performed using a JEOL JSM6700F scanning electron microscope as described previously [63]. For thin sections, square 2 mm blocks were cut from 6-day-old cultures and fixed with 1.5% glutaraldehyde in 0.1 M cacodylate buffer for 1 h, rinsed with 0.1 M cacodylate buffer, and post-fixed with osmium tetroxide for 1 h. Subsequently, samples were dehydrated (70% ethanol 15 min, 80% ethanol 15 min, 90% ethanol 15 min, 100% ethanol 15 min, 100% propylene oxide 1 h). Subsequently, samples were embedded in Epon resin by infiltrating for 1 h in a 1 : 1 Epon : propylene oxide mixture followed by 2 days incubation at 60°C for Epon polymerization. Sections (70 nm) were prepared and stained with uranyl acetate (10 min) and lead citrate (10 min). TEM for the analysis of cross sections of hyphae and spores was performed with a FEI Tecnai 12 BioTwin transmission electron microscope [64].

Acceptor photobleaching was performed with a Zeiss Imager. eGFP was excited with a 488 nm laser (5% intensity), and emission was detected from 505 to 530 nm. mCherry was excited with 543 nm laser (5% intensity) and detected with a 560 long-pass. Both channels were recorded sequentially to prevent signal cross-bleeding. Bleaching was performed with the 543 nm laser at maximum intensity for 25 iterations. For all experiments, the original intensity was determined as the average of three pre-bleach frames, and the post-bleaching intensity was set as the average of the first three post-bleach frames. For SsgA–eGFP with SepG–mCherry (no interaction), 11 independent experiments were carried out, for FtsZ–mCherry and SepG–eGFP 18, and for SsgB–eGFP and SepG–mCherry 24 (table 2). The average post-bleach intensity was calculated relative to the pre-bleach intensity, including standard error. All images were collected in a 512 × 512 pixel format with a 63 × 1.4 NA oil objective.

For FRAP experiments, post-bleach intensity was followed for 15 s. Intensity measurements were corrected for image bleaching by examining the loss of fluorescence intensity in non-bleached regions. Images and bleaches were performed with similar settings as for the FRET experiments.

### 4.4. Heat resistance test

Fresh spores were obtained from SFM agar plates after 5 days of incubation at 30°C. Spore suspensions were diluted in water and heat treated at 60°C for 10 min. Dilutions were plated on SFM agar plates, incubated at 30°C and survival rates determined. Experiments were carried out in triplicate.

### 4.5. Membrane topology analysis of SepG

*Escherichia coli* JM109 cells were transformed with constructs expressing different BlaM-fusion proteins. Transformants were grown in 1 ml LB media containing apramycin 50 µg ml^−1^ for 4 h to reach OD_600_ 0.4–0.6. Cells were washed twice with LB to remove accumulated β-lactamases and resuspended in 1 ml LB + 10% glycerol. 0.5 µl cell suspension was spotted onto LB agar plates with different concentrations of ampicillin, and growth was observed after overnight incubation at 37°C. To investigate the growth rate of JM109 carrying different constructs, 3 µl of prewashed cell suspension was inoculated into 200 µl LB with or without ampicillin. The optical density was measured every 30 min for 12 h using an automated Bioscreen optical plate reader (Oy Growth Curves Ab Ltd, Finland), with settings of 37°C, continuous shaking, OD wideband filter and a start OD programmatically set to 0.100 for easier comparison. All samples were carried out using three independent transformants, and each of these was analysed in triplicate.

### 4.6. Computer analysis

Analysis of *Streptomyces* genes and proteins was done at the StrepDB database (http://strepdb.streptomyces.org.uk/). For phylogenetic analysis and correlations, the String engine (http://string.embl.de) was used, whereas putative transmembrane domains were identified using transmembrane prediction server DAS [65]. The topological model was generated using program TMRPRES2D [47]. Amino acid sequence alignment was done using Clustal Omega (http://www.ebi.ac.uk/Tools/msa/clustalo).

## Supplementary Material

Supplementary Tables

## References

[1] Bi EF, Lutkenhaus J 1991 FtsZ ring structure associated with division in *Escherichia coli*. Nature 354, 161–164. (doi:10.1038/354161a0) 1944597 10.1038/354161a0

[2] Goehring NW, Beckwith J 2005 Diverse paths to midcell: assembly of the bacterial cell division machinery. Curr. Biol. 15, R514–R526. (doi:10.1016/j.cub.2005.06.038) 16005287 10.1016/j.cub.2005.06.038

[3] Adams DW, Errington J 2009 Bacterial cell division: assembly, maintenance and disassembly of the Z ring. Nat. Rev. Microbiol. 7, 642–653. (doi:10.1038/nrmicro2198) 19680248 10.1038/nrmicro2198

[4] Hale CA, de Boer PAJ 1997 Direct binding of FtsZ to ZipA, an essential component of the septal ring structure that mediates cell division in *E. coli*. Cell 88, 175–185. (doi:10.1016/S0092-8674(00)81838-3) 9008158 10.1016/s0092-8674(00)81838-3

[5] RayChaudhuri D 1999 ZipA is a MAP-Tau homolog and is essential for structural integrity of the cytokinetic FtsZ ring during bacterial cell division. EMBO J. 18, 2372–2383. (doi:10.1093/emboj/18.9.2372) 10228152 10.1093/emboj/18.9.2372 PMC1171320

[6] Pichoff S, Lutkenhaus J 2002 Unique and overlapping roles for ZipA and FtsA in septal ring assembly in *Escherichia coli*. EMBO J. 21, 685–693. (doi:10.1093/emboj/21.4.685) 11847116 10.1093/emboj/21.4.685 PMC125861

[7] Gueiros-Filho FJ, Losick R 2002 A widely conserved bacterial cell division protein that promotes assembly of the tubulin-like protein FtsZ. Genes Dev. 16, 2544–2556. (doi:10.1101/gad.1014102) 12368265 10.1101/gad.1014102 PMC187447

[8] Hamoen LW, Meile JC, de Jong W, Noirot P, Errington J 2006 SepF, a novel FtsZ-interacting protein required for a late step in cell division. Mol. Microbiol. 59, 989–999. (doi:10.1111/j.1365-2958.2005.04987.x) 16420366 10.1111/j.1365-2958.2005.04987.x

[9] Ishikawa S, Kawai Y, Hiramatsu K, Kuwano M, Ogasawara N 2006 A new FtsZ-interacting protein, YlmF, complements the activity of FtsA during progression of cell division in *Bacillus subtilis*. Mol. Microbiol. 60, 1364–1380. (doi:10.1111/j.1365-2958.2006.05184.x) 16796675 10.1111/j.1365-2958.2006.05184.x

[10] Romberg L, Levin PA 2003 Assembly dynamics of the bacterial cell division protein FtsZ: poised at the edge of stability. Annu. Rev. Microbiol. 57, 125–154. (doi:10.1146/annurev.micro.57.012903.074300) 14527275 10.1146/annurev.micro.57.012903.074300 PMC5517307

[11] Claessen D, Rozen DE, Kuipers OP, Sogaard-Andersen L, van Wezel GP 2014 Bacterial solutions to multicellularity: a tale of biofilms, filaments and fruiting bodies. Nat. Rev. Microbiol. 12, 115–124. (doi:10.1038/nrmicro3178) 24384602 10.1038/nrmicro3178

[12] Flärdh K, Buttner MJ 2009 *Streptomyces* morphogenetics: dissecting differentiation in a filamentous bacterium. Nat. Rev. Microbiol. 7, 36–49. (doi:10.1038/nrmicro1968) 19079351 10.1038/nrmicro1968

[13] Barka EA, Vatsa P, Sanchez L, Gavaut-Vaillant N, Jacquard C, Klenk HP, Clément C, Oudouch Y, van Wezel GP 2016 Taxonomy, physiology, and natural products of the *Actinobacteria*. Microbiol. Mol. Biol. Rev. 80, 1–43. (doi:10.1128/MMBR.masthead.80-1) 26609051 10.1128/MMBR.00019-15 PMC4711186

[14] Hopwood DA 2007 Streptomyces in nature and medicine: the antibiotic makers. New York, NY: Oxford University Press.

[15] van Wezel GP, McDowall KJ 2011 The regulation of the secondary metabolism of *Streptomyces*: new links and experimental advances. Nat. Prod. Rep. 28, 1311–1333. (doi:10.1039/c1np00003a) 21611665 10.1039/c1np00003a

[16] Wildermuth H, Hopwood D 1970 Septation during sporulation in *Streptomyces coelicolor*. J. Gen. Microbiol. 60, 51–59. (doi:10.1099/00221287-60-1-51) 5488466 10.1099/00221287-60-1-51

[17] Schwedock J, McCormick JR, Angert ER, Nodwell JR, Losick R 1997 Assembly of the cell division protein FtsZ into ladder like structures in the aerial hyphae of *Streptomyces coelicolor*. Mol. Microbiol. 25, 847–858. (doi:10.1111/j.1365-2958.1997.mmi507.x) 9364911 10.1111/j.1365-2958.1997.mmi507.x

[18] Jakimowicz D, van Wezel GP 2012 Cell division and DNA segregation in *Streptomyces*: how to build a septum in the middle of nowhere? Mol. *Microbiol* 85, 393–404. 10.1111/j.1365-2958.2012.08107.x 22646484

[19] McCormick JR, Losick R 1996 Cell division gene ftsQ is required for efficient sporulation but not growth and viability in *Streptomyces coelicolor* A3(2). J. Bacteriol. 178, 5295–5301. 8752351 10.1128/jb.178.17.5295-5301.1996 PMC178330

[20] McCormick JR, Su EP, Driks A, Losick R 1994 Growth and viability of *Streptomyces coelicolor* mutant for the cell division gene *ftsZ*. Mol. Microbiol. 14, 243–254. (doi:10.1111/j.1365-2958.1994.tb01285.x) 7830569 10.1111/j.1365-2958.1994.tb01285.x

[21] Marston AL, Thomaides HB, Edwards DH, Sharpe ME, Errington J 1998 Polar localization of the MinD protein of *Bacillus subtilis* and its role in selection of the mid-cell division site. Genes Dev. 12, 3419–3430. (doi:10.1101/gad.12.21.3419) 9808628 10.1101/gad.12.21.3419 PMC317235

[22] Raskin DM, de Boer PAJ 1997 The MinE ring: an FtsZ-independent cell structure required for selection of the correct division site in *E. coli*. Cell 91, 685–694. (doi:10.1016/S0092-8674(00)80455-9) 9393861 10.1016/s0092-8674(00)80455-9

[23] Bernhardt TG, de Boer PAJ 2005 SlmA, a nucleoid-associated, FtsZ binding protein required for blocking septal ring assembly over chromosomes in *E. coli*. Mol. Cell 18, 555–564. (doi:10.1016/j.molcel.2005.04.012) 15916962 10.1016/j.molcel.2005.04.012 PMC4428309

[24] Woldringh CL, Mulder E, Huls PG, Vischer N 1991 Toporegulation of bacterial division according to the nucleoid occlusion model. Res. Microbiol. 142, 309–320. (doi:10.1016/0923-2508(91)90046-D) 1925029 10.1016/0923-2508(91)90046-d

[25] Wu LJ, Errington J 2004 Coordination of cell division and chromosome segregation by a nucleoid occlusion protein in *Bacillus subtilis*. Cell 117, 915–925. (doi:10.1016/j.cell.2004.06.002) 15210112 10.1016/j.cell.2004.06.002

[26] Wu LJ, Errington J 2012 Nucleoid occlusion and bacterial cell division. Nat. Rev. Microbiol. 10, 8–12. 22020262 10.1038/nrmicro2671

[27] Willemse J, Borst JW, de Waal E, Bisseling T, van Wezel GP 2011 Positive control of cell division: FtsZ is recruited by SsgB during sporulation of *Streptomyces*. Genes Dev. 25, 89–99. (doi:10.1101/gad.600211) 21205868 10.1101/gad.600211 PMC3012939

[28] Traag BA, van Wezel GP 2008 The SsgA-like proteins in actinomycetes: small proteins up to a big task. Antonie Van Leeuwenhoek 94, 85–97. (doi:10.1007/s10482-008-9225-3) 18273689 10.1007/s10482-008-9225-3 PMC2440963

[29] van Dissel D, Claessen D, Van Wezel GP 2014 Morphogenesis of *Streptomyces* in submerged cultures. Adv. Appl. Microbiol. 89, 1–45. (doi:10.1016/B978-0-12-800259-9.00001-9) 25131399 10.1016/B978-0-12-800259-9.00001-9

[30] Flärdh K 2003 Essential role of DivIVA in polar growth and morphogenesis in *Streptomyces coelicolor* A3(2). Mol. Microbiol. 49, 1523–1536. (doi:10.1046/j.1365-2958.2003.03660.x) 12950918 10.1046/j.1365-2958.2003.03660.x

[31] Treuner-Lange A et al. 2013 PomZ, a ParA-like protein, regulates Z-ring formation and cell division in *Myxococcus xanthus*. Mol. Microbiol. 87, 235–253. (doi:10.1111/mmi.12094) 23145985 10.1111/mmi.12094

[32] Rodrigues CD, Harry EJ 2012 The Min system and nucleoid occlusion are not required for identifying the division site in *Bacillus subtilis* but ensure its efficient utilization. PLoS Genet. 8, e1002561 (doi:10.1371/journal.pgen.1002561) 22457634 10.1371/journal.pgen.1002561 PMC3310732

[33] Edwards DH, Errington J 1997 The *Bacillus subtilis* DivIVA protein targets to the division septum and controls the site specificity of cell division. Mol. Microbiol. 24, 905–915. (doi:10.1046/j.1365-2958.1997.3811764.x) 9219999 10.1046/j.1365-2958.1997.3811764.x

[34] Ito T, Uozumi N, Nakamura T, Takayama S, Matsuda N, Aiba H, Hemmi H, Yoshimura T 2009 The implication of YggT of *Escherichia coli* in osmotic regulation. Biosci. Biotechnol. Biochem. 73, 2698–2704. (doi:10.1271/bbb.90558) 19966467 10.1271/bbb.90558

[35] Kabeya Y, Nakanishi H, Suzuki K, Ichikawa T, Kondou Y, Matsui M, Miyagishima S-y 2010 The YlmG protein has a conserved function related to the distribution of nucleoids in chloroplasts and cyanobacteria. BMC Plant Biol. 10, 57 (doi:10.1186/1471-2229-10-57) 20359373 10.1186/1471-2229-10-57 PMC2923531

[36] Marbouty M, Saguez C, Cassier-Chauvat C, Chauvat F 2009 ZipN, an FtsA-like orchestrator of divisome assembly in the model cyanobacterium *Synechocystis* PCC6803. Mol. Microbiol. 74, 409–420. (doi:10.1111/j.1365-2958.2009.06873.x) 19737354 10.1111/j.1365-2958.2009.06873.x

[37] Fadda D, Pischedda C, Caldara F, Whalen MB, Anderluzzi D, Domenici E, Massidda O 2003 Characterization of divIVA and other genes located in the chromosomal region downstream of the dcw cluster in *Streptococcus pneumoniae*. J. Bacteriol. 185, 6209–6214. (doi:10.1128/JB.185.20.6209-6214.2003) 14526035 10.1128/JB.185.20.6209-6214.2003 PMC225046

[38] Girard G, Traag BA, Sangal V, Mascini N, Hoskisson PA, Goodfellow M, van Wezel GP 2013 A novel taxonomic marker that discriminates between morphologically complex actinomycetes. Open Biol. 3, 130073 (doi:10.1098/rsob.130073) 24153003 10.1098/rsob.130073 PMC3814722

[39] Duman R, Ishikawa S, Celik I, Strahl H, Ogasawara N, Troc P, Löwe J, Hamoen LW 2013 Structural and genetic analyses reveal the protein SepF as a new membrane anchor for the Z ring. Proc. Natl Acad. Sci. USA 110, E4601–E4610. (doi:10.1073/pnas.1313978110) 24218584 10.1073/pnas.1313978110 PMC3845145

[40] Pogliano K, Harry E, Losick R 1995 Visualization of the subcellular location of sporulation proteins in *Bacillus subtilis* using immunofluorescence microscopy. Mol. Microbiol. 18, 459–470. (doi:10.1111/j.1365-2958.1995.mmi_18030459.x) 8748030 10.1111/j.1365-2958.1995.mmi_18030459.x

[41] Flärdh K, Leibovitz E, Buttner MJ, Chater KF 2000 Generation of a non-sporulating strain of *Streptomyces coelicolor* A3(2) by the manipulation of a developmentally controlled ftsZ promoter. Mol. Microbiol. 38, 737–749. (doi:10.1046/j.1365-2958.2000.02177.x) 11115109 10.1046/j.1365-2958.2000.02177.x

[42] Swiatek-Polatynska MA, Bucca G, Laing E, Gubbens J, Titgemeyer F, Smith CP, Rigali S, van Wezel GP 2015 Genome-wide analysis of *in vivo* binding of the master regulator DasR in *Streptomyces coelicolor* identifies novel non-anonical targets. PLoS ONE 10, e0122479 (doi:10.1371/journal.pone.0122479) 25875084 10.1371/journal.pone.0122479 PMC4398421

[43] Mullineaux CW, Nenninger A, Ray N, Robinson C 2006 Diffusion of green fluorescent protein in three cell environments in *Escherichia coli*. J. Bacteriol. 188, 3442–3448. (doi:10.1128/JB.188.10.3442-3448.2006) 16672597 10.1128/JB.188.10.3442-3448.2006 PMC1482841

[44] Broome-Smith JK, Tadayyon M, Zhang Y 1990 Beta-lactamase as a probe of membrane protein assembly and protein export. Mol. Microbiol. 4, 1637–1644. (doi:10.1111/j.1365-2958.1990.tb00540.x) 2077355 10.1111/j.1365-2958.1990.tb00540.x

[45] Tadayyon M, Zhang Y, Gnaneshan S, Hunt L, Mehraein-Ghomi F, Broome-Smith JK 1992 Beta-lactamase fusion analysis of membrane protein assembly. Biochem. Soc. Trans. 20, 598–601. (doi:10.1042/bst0200598) 1426596 10.1042/bst0200598

[46] Mueller KE, Fields KA 2015 Application of beta-lactamase reporter fusions as an indicator of effector protein secretion during infections with the obligate intracellular pathogen *Chlamydia trachomatis*. PLoS ONE 10, e0135295 (doi:10.1371/journal.pone.0135295) 26258949 10.1371/journal.pone.0135295 PMC4530969

[47] Spyropoulos IC, Liakopoulos TD, Bagos PG, Hamodrakas SJ 2004 TMRPres2D: high quality visual representation of transmembrane protein models. Bioinformatics 20, 3258–3260. (doi:10.1093/bioinformatics/bth358) 15201184 10.1093/bioinformatics/bth358

[48] Swiatek MA, Tenconi E, Rigali S, van Wezel GP 2012 Functional analysis of the N-acetylglucosamine metabolic genes of *Streptomyces coelicolor* and role in the control of development and antibiotic production. J. Bacteriol. 194, 1136–1144. (doi:10.1128/JB.06370-11) 22194457 10.1128/JB.06370-11 PMC3294797

[49] Ragkousi K, Cowan AE, Ross MA, Setlow P 2000 Analysis of nucleoid morphology during germination and outgrowth of spores of *Bacillus* species. J. Bacteriol. 182, 5556–5562. (doi:10.1128/JB.182.19.5556-5562.2000) 10986261 10.1128/jb.182.19.5556-5562.2000 PMC111001

[50] Sambrook J, Fritsch E, Maniatis T 1989 Molecular cloning: a laboratory manual. New York, NY: Cold Spring Harbor Laboratory Press.

[51] MacNeil DJ, Gewain KM, Ruby CL, Dezeny G, Gibbons PH, Maeneil T 1992 Analysis of *Streptomyces avermitilis* genes required for avermectin biosynthesis utilizing a novel integration vector. Gene 111, 61–68. (doi:10.1016/0378-1119(92)90603-M) 1547955 10.1016/0378-1119(92)90603-m

[52] Kieser T, Bibb MJ, Buttner MJ, Chater KF, Hopwood DA 2000 Practical *Streptomyces* genetics. Norwich, UK: John Innes Foundation.

[53] van Wezel GP, van der Meulen J, Kawamoto S, Luiten RG, Koerten HK, Kraal B 2000 ssgA is essential for sporulation of *Streptomyces coelicolor* A3(2) and affects hyphal development by stimulating septum formation. J. Bacteriol. 182, 5653–5662. (doi:10.1128/JB.182.20.5653-5662.2000) 11004161 10.1128/jb.182.20.5653-5662.2000 PMC94684

[54] Keijser BJ, Noens EE, Kraal B, Koerten HK, van Wezel GP 2003 The *Streptomyces coelicolor ssgB* gene is required for early stages of sporulation. FEMS Microbiol. Lett. 225, 59–67. (doi:10.1016/S0378-1097(03)00481-6) 12900022 10.1016/S0378-1097(03)00481-6

[55] Colson S, Stephan J, Hertrich T, Saito A, van Wezel GP, Titgemeyer F, Rigali S 2007 Conserved *cis*-acting elements upstream of genes composing the chitinolytic system of streptomycetes are DasR-responsive elements. J. Mol. Microbiol. Biotechnol. 12, 60–66. (doi:10.1159/000096460) 17183212 10.1159/000096460

[56] Vara J, Lewandowska-Skarbek M, Wang YG, Donadio S, Hutchinson CR 1989 Cloning of genes governing the deoxysugar portion of the erythromycin biosynthesis pathway in *Saccharopolyspora erythraea* (*Streptomyces erythreus*). J. Bacteriol. 171, 5872–5881. 2681144 10.1128/jb.171.11.5872-5881.1989 PMC210448

[57] Fedoryshyn M, Welle E, Bechthold A, Luzhetskyy A 2008 Functional expression of the Cre recombinase in actinomycetes. Appl. Microbiol. Biotechnol. 78, 1065–1070. (doi:10.1007/s00253-008-1382-9) 18299828 10.1007/s00253-008-1382-9

[58] Bierman M, Logan R, O'Brien K, Seno ET, Nagaraja Rao R, Schoner BE 1992 Plasmid cloning vectors for the conjugal transfer of DNA from *Escherichia coli* to *Streptomyces* spp. Gene 116, 43–49. (doi:10.1016/0378-1119(92)90627-2) 1628843 10.1016/0378-1119(92)90627-2

[59] Grantcharova N, Lustig U, Flärdh K 2005 Dynamics of FtsZ assembly during sporulation in *Streptomyces coelicolor* A3(2). J. Bacteriol. 187, 3227–3237. (doi:10.1128/JB.187.9.3227-3237.2005) 15838050 10.1128/JB.187.9.3227-3237.2005 PMC1082811

[60] Larson JL, Hershberger CL 1986 The minimal replicon of a streptomycete plasmid produces an ultrahigh level of plasmid DNA. Plasmid 15, 199–209. (doi:10.1016/0147-619X(86)90038-7) 3012613 10.1016/0147-619x(86)90038-7

[61] van Wezel GP, White J, Hoogvliet G, Bibb MJ 2000 Application of redD, the transcriptional activator gene of the undecylprodigiosin biosynthetic pathway, as a reporter for transcriptional activity in *Streptomyces coelicolor* A3(2) and *Streptomyces lividans*. J. Mol. Microbiol. Biotechnol. 2, 551–556. 11075931

[62] Willemse J, van Wezel GP 2009 Imaging of *Streptomyces coelicolor* A3(2) with reduced autofluorescence reveals a novel stage of FtsZ localization. PLoS ONE 4, e4242 (doi:10.1371/journal.pone.0004242) 19156202 10.1371/journal.pone.0004242 PMC2625393

[63] Colson S, van Wezel GP, Craig M, Noens EEE, Nothaft H, Mommaas AM, Titgemeyer F, Joris B, Rigali S 2008 The chitobiose-binding protein, DasA, acts as a link between chitin utilization and morphogenesis in *Streptomyces coelicolor*. Microbiology 154, 373–382. (doi:10.1099/mic.0.2007/011940-0) 18227241 10.1099/mic.0.2007/011940-0

[64] Piette A et al. 2005 From dormant to germinating spores of *Streptomyces coelicolor* A3(2): new perspectives from the *crp* null mutant. J. Proteome Res. 4, 1699–1708. (doi:10.1021/pr050155b) 16212423 10.1021/pr050155b

[65] Cserzo M, Eisenhaber F, Eisenhaber B, Simon I 2004 TM or not TM: transmembrane protein prediction with low false positive rate using DAS-TMfilter. Bioinformatics 20, 136–137. (doi:10.1093/bioinformatics/btg394) 14693825 10.1093/bioinformatics/btg394

