# Supplementary material for: SepG coordinates sporulation-specific cell division and nucleoid organization in Streptomyces coelicolor
Source: Open Biol. 2016 Apr 6;6(4):150164. doi: 10.1098/rsob.150164 (PMC4852450; doi:10.1098/rsob.150164)
Supplement: Supplementary Tables [file rsob150164supp1.pdf]

SUPPLEMENTAL INFORMATION

Belonging to the manuscript:

SepG coordinates sporulation-specific cell division and nucleoid organization in *Streptomyces coelicolor*

Le Zhang*, Joost Willemse*, Dennis Claessen and Gilles P. van Wezel[#]

Molecular Biotechnology, Institute of Biology, Leiden University, PO Box 9505, 2300RA, Leiden, The Netherlands

* These authors contributed equally to this work

[#]To whom correspondence should be addressed. Tel: +3171527430; email: g.wezel@biology.leidenuniv.nl

Table S1. Bacterial strains**.**

**Bacterial strains**	**Genotype**	**Reference**
E. coli JM109	See reference	[1]
E. coli ET12567	See reference	[2]
M145	*S. coelicolor* A3(2) SCP1- SCP2-	[3]
K202	M145 + KF41	[4]
GAL1	M145Δ*ylmG*	This work
GAL2	M145 + pGWS755	This work
GAL3	GAL1 + KF41	This work
GAL4	GAL1 + pGWS116	This work
GAL5	GAL1 + pGWS526	This work
GAL7	GAL1 + pGWS755	This work
GSA3	M145Δ*ssgA*(*::aadA*)	[5]
GSB1	M145Δ*ssgB*(::*aac(3)IV*)	[6]
GAL93	GAL1 + pGWS771	This work
GAL94	M145 + pGWS791 + pGWS116	This work
GAL95	M145 + pGWS791 + pGWS526	This work
GAL96	M145 + pGWS755 + pGWS529	This work
GAL102	M145 + pGWS526 + pGWS527	This work
GAL103	M145 + pGWS755 + GAPp-*mCherry*	This work

Table S2. Plasmids and constructs.

**Plasmid and constructs**	**Description**	**Reference**
pGWS116	pHJL401 harbouring SsgA-eGFP	[7]
pGWS526	pHJL401 harbouring SsgB-eGFP	[8]
pGWS527	pSET152 harbouring FtsZ-mCherry behind the native*ftsZ* promoter	This work
pGWS529	pHJL401 expressing FtsZ-mCherry from the *S. coelicolor ftsZ* promoter.	This work
pGWS725	pWHM3 with XbaI site removed from the multiple cloning site.	This work
pGWS731	pWHM3 containing around 1.5 kb flanking regions of *S. coelicolor* SCO2078 with apra-*loxP* inserted in between.	This work
pGWS755	pSET152 harbouring the *ylmG-egfp* under the control of the *S. coelicolor ftsZ* promoter region	This work
pGWS756	pHJL401 harbouring the *ylmG-egfp* under the control of the *S. coelicolor ftsZ* promoter region	This work
pGWS771	pSET152 harbouring the +1/+294 region of *ylmG* under the control of the *ftsZ* promoter region of *S. coelicolor*.	This work
pGWS791	pSET152 with *ylmG-mCherry* under the control of the *ftsZ* promoter region of *S. coelicolor*	This work
pGWS793	pSET152 harbouring *sepG-blaM* under the control of the *S. coelicolor ftsZ* promoter region	This work
pGWS794	As pGWS794 but expressing the N-terminal part of SepG fused to BlaM	This work
pGWS795	As pGWS794 but expressing the C-terminal part of SepG fused to BlaM	This work
pGWS796	As pGWS794 but expressing full-length BlaM	This work
pGWS797	As pGWS794 but expressing mature BlaM (no signal peptide)	This work
pHJL401	*E. coli*/*Streptomyces* shuttle vector, low copy (<5 copies per chromosome) in *Streptomyces* and high copy (>100 copies per chromosome) in *E. coli*	[9]
pKF41	Integrative construct based on pSET152 expressing *ftsZ-egfp* from the *S. coelicolor ftsZ* promoter region	[4]
pKT25	BACTH plasmid harboring the T18 fragment of	[10]

	the<br>Bordetella pertussis *cya* gene.	
pSET152	*E. coli*/*Streptomyces* shuttle vector, around 100 copies per chromosome in *E. coli* and integrative in the ϕC31 attachment site in *Streptomyces*	[11]
pUWL-Cre	Plasmid harboring Cre-recombinase	[12]
pWHM3	*E. coli*/*Streptomyces* shuttle vector, around 100 copies per chromosome in both *E. coli* and *Streptomyces*.	[13]

Table S3. Oligonucleotides.

**Name**	**5’-3’ sequence #**
ylmG_LF-1442	GTCA**GAATTC**GCGGACGATGTGCGCATTCTCTCG
ylmG_LR+6	GTCAGAAGTTATCCATCACC**TCTAGA**GCTCATGACCTGTGCTTCCCTCTC
ylmG_RF+277	GTCAGAAGTTATCGCGCATC**TCTAGA**CAGCTGTGAGCGATGAGAGAGATAC
ylmG_RR+1521	GTCA**GGATCC**TCGATCAGGAACCCGCGCATCGGC
ylmG_F+1	GTCAGAATTC**AGGCCT**TCGACATGAGCGTGGTCCTGGATGTC
ylmG_R+282	GTCAAAGCTT**GGATCC**AGCTGGCTCACGATCGAGAT
ylmG_R+294	GCTA**GGATCC**TCTCATCGCTCACAGCTGGCT
BlaM_F_EB	ctgaGAATTC**GGATCC**GGCGGTTCCACATGCTATAAGTTCCAGCCCATTACGCCACCCAGAAACGCTGGTGAAA
BlaM_R_XH	ctgaAAGCTT**TCTAGA**TTACCAATGCTTAATCAGTGAGGC
YlmG_R+120	ctgaAAGCTT**GGATCC**GCCTTGCCGGGTTGC
YlmG_F+121	ctgaGAATTC**AGGCCT**TCGACATGGTGGTCGTTCTGGAGGCCA
BlaM_F+1_ES	ctgaGAATTC**AGGCCT**TCGACATGAGTATTCAACATTTCCGT
BlaM_F+70_ES	ctgaGAATTC**AGGCCT**TCGACATGCACCCAGAAACGCTGGTGAAA

Restriction sites used for cloning are underlined and in bold face. GGATCC, BamHI; GAATTC, EcoRI; AGGCCT, StuI; TCTAGA, XbaI; AAGCTT, HindIII. Sequence shadowed in grey encoded the 12 amino acids linker between SepG and BlaM or FtsZ and BlaM

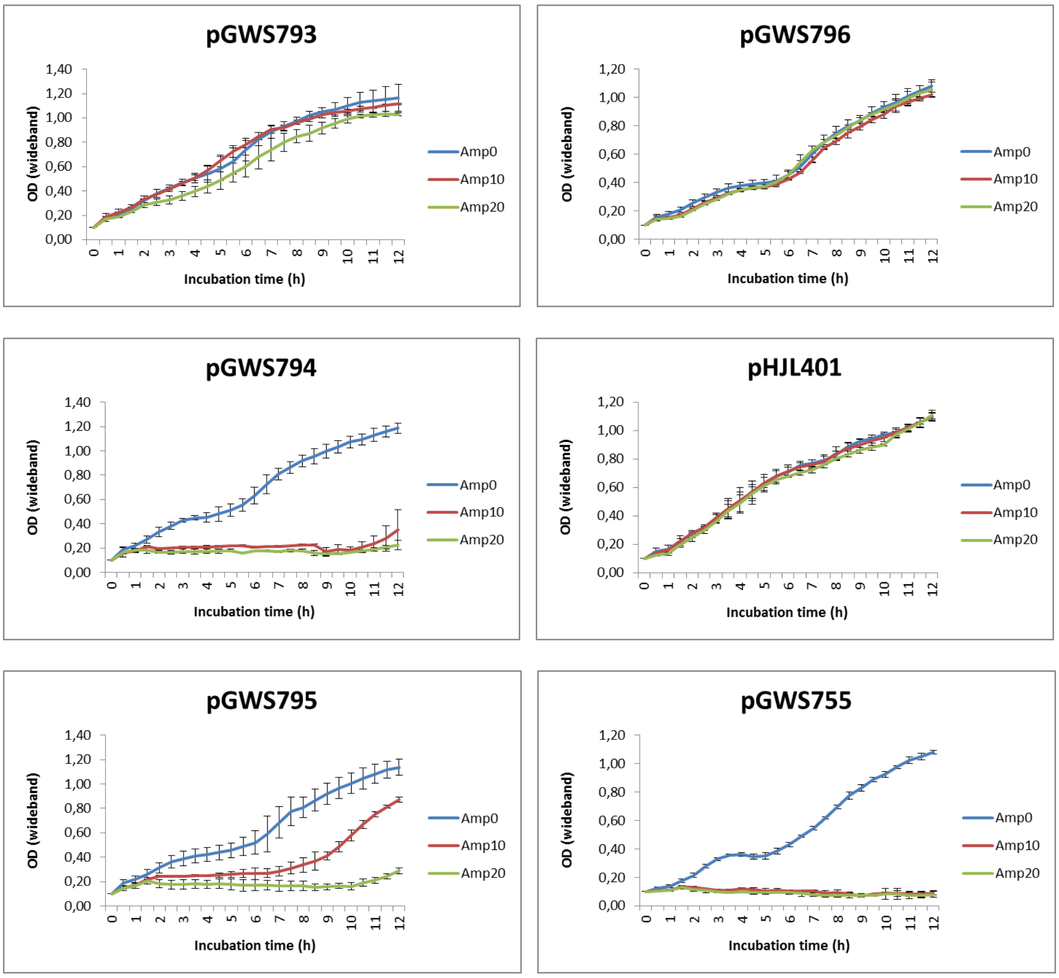

Figure S1. Growth curves of *E. coli* JM109 carrying plasmids expressing different BlaM fusions. The graphs belong to Figure 8 of the manuscript. The BlaM fusions were: full-length SepG-BlaM (pGWS793); N-terminal SepG$_{1-40}$-BlaM (pGWS794; first TM domain of SepG); C-terminal SepG$_{41-94}$-BlaM (pGWS795; second TM domain of SepG); BlaM with signal sequence expressed from the ftsZ promoter(s) (pGWS796; positive control); BlaM with signal sequence expressed from its native promoter (pHJL401; positive control); and pGWS755 expressing SepG-eGFP (pGWS755; negative control). Transformants were grown in presence of 0, 10 or 20 µg/ml ampicillin. Growth curve for each strain was measured every 30 minutes for 12 h at 37^{0}C. Cultures were obtained for three independent transformants (and each of these in triplicate) using a Bioscreen C microtitre system with automated biomass reading.

REFERENCES TO SUPPLEMENTAL DATA

1. Sambrook J, Fritsch E, Maniatis T. 1989 *Molecular cloning: a laboratory manual.*: New York: Cold spring harbor laboratory press.

2. MacNeil DJ, Gewain KM, Ruby CL, Dezeny G, Gibbons PH, Maeneil T. 1992 Analysis of *Streptomyces avermitilis* genes required for avermectin biosynthesis utilizing a novel integration vector. *Gene*. **111**, 61-68.

3. Kieser T, Bibb MJ, Buttner MJ, Chater KF, Hopwood DA. 2000 *Practical Streptomyces genetics.*: John Innes Foundation.

4. Grantcharova N, Lustig U, Flärdh K. 2005 Dynamics of FtsZ assembly during sporulation in *Streptomyces coelicolor* A3(2). *Journal of bacteriology*. **187**, 3227-3237.

5. van Wezel GP, van der Meulen J, Kawamoto S, Luiten RG, Koerten HK, Kraal B. 2000 SsgA is essential for sporulation of *Streptomyces coelicolor* A3(2) and affects hyphal development by stimulating septum formation. *Journal of bacteriology*. **182**, 5653-5662.

6. Keijser BJF, Noens EE, Kraal B, Koerten HK, van Wezel GP. 2003 The *Streptomyces coelicolor ssgB* gene is required for early stages of sporulation. *FEMS Microbiol. Lett.* **225**, 59-67.

7. Noens EE, Mersinias V, Willemse J, Traag BA, Laing E, Chater KF, Smith CP, Koerten HK, van Wezel GP. 2007 Loss of the controlled localization of growth stage-specific cell-wall synthesis pleiotropically affects developmental gene expression in an *ssgA* mutant of *Streptomyces coelicolor*. *Mol. Microbiol.* **64**, 1244-1259.

8. Willemse J, Borst JW, de Waal E, Bisseling T, van Wezel GP. 2011 Positive control of cell division: FtsZ is recruited by SsgB during sporulation of *Streptomyces*. *Genes Dev*. **25**, 89-99.

9. Larson JL, Hershberger CL. 1986 The minimal replicon of a streptomycete plasmid produces an ultrahigh level of plasmid DNA. *Plasmid*. **15**, 199-209.

10. Karimova G, Pidoux J, Ullmann A, Ladant D. 1998 A bacterial two-hybrid system based on a reconstituted signal transduction pathway. *Proc. Natl. Acad. Sci. U. S. A.* **95**, 5752-5756.

11. Bierman M, Logan R, O'Brien K, Seno ET, Nagaraja Rao R, Schoner BE. 1992 Plasmid cloning vectors for the conjugal transfer of DNA from *Escherichia coli* to *Streptomyces* spp. *Gene*. **116**, 43-49.

12. Fedoryshyn M, Welle E, Bechthold A, Luzhetskyy A. 2008 Functional expression of the Cre recombinase in actinomycetes. *Appl. Microbiol. Biotechnol.* **78**, 1065-1070.

13. Vara J, Lewandowska-Skarbek M, Wang YG, Donadio S, Hutchinson CR. 1989 Cloning of genes governing the deoxysugar portion of the erythromycin biosynthesis pathway in *Saccharopolyspora erythraea* (*Streptomyces erythreus*). *J. Bacteriol.* **171**, 5872-5881.
